# Therapeutic Potential of Bovine Colostrum- and Milk-Derived Exosomes in Cancer Prevention and Treatment: Mechanisms, Evidence, and Future Perspectives

**DOI:** 10.3390/ph19010168

**Published:** 2026-01-17

**Authors:** Yusuf Serhat Karakülah, Yalçın Mert Yalçıntaş, Mikhael Bechelany, Sercan Karav

**Affiliations:** 1 Department of Molecular Biology and Genetics, Çanakkale Onsekiz Mart University, Çanakkale 17000, Turkey; yusufserhatkarakulah@gmail.com (Y.S.K.); yalcinmertyalcintas@stu.comu.edu.tr (Y.M.Y.); 2 European Institute for Membranes (IEM), UMR-5635, University of Montpellier, École Nationale Supérieure de Chimie de Montpellier (ENSCM), Centre National de la Recherche Scientifique (CNRS), Place Eugène Bataillon, CEDEX 5, F-34095 Montpellier, France; 3 Department of Medical Research, China Medical University Hospital, China Medical University, Taichung City 40447, Taiwan

**Keywords:** exosomes, nanocarriers, anticancer, delivery, apoptosis

## Abstract

Due to their therapeutic potential and effects on cells, exosomes derived from bovine colostrum (BCE) and milk (BME) are molecules that have been at the center of recent studies. Their properties include the ability to cross biological barriers, their natural biocompatibility, and their structure, which enable them to act as stable nanocarriers. Exosomes derived from milk and colostrum stand out in cancer prevention and treatment due to these properties. BMEs can be enriched with bioactive peptides, lipids, and nucleic acids. The targeted drug delivery capacity of BMEs can be made more efficient through these enrichment processes. For example, BME enriched with an iRGD peptide and developed using hypoxia-sensitive lipids selectively transported drugs and reduced the survival rate of triple-negative breast cancer (TNBC) cells. ARV-825-CME formulations increased antitumor activity in some cancer types. The anticancer effects of exosomes are supported by these examples. In addition to their anticancer activities, exosomes also exhibit effects that maintain immune balance. BME and BCE can regulate inflammatory responses with their miRNA and protein loads. These effects of BMEs have been demonstrated in studies on colon, breast, liver, and lung cancers. The findings support the safety and scalability of these effects. However, significant challenges remain in terms of their large-scale isolation, load heterogeneity, and regulatory standardization. Consequently, BMEs represent a new generation of biogenic nanoplatforms at the intersection of nutrition, immunology, and oncology, paving the way for innovative therapeutic approaches.

## 1. Introduction

Milk is an essential nutrient due to its rich bioactive composition. Along with this, colostrum forms in the first days after birth. It contains higher levels of immune and bioactive compounds than those found in mature milk [1]. Bovine colostrum (BC) is also rich in these ingredients. Bioactive components enable BC to support the development of passive immunity in newborn calves. Low or inadequate BC intake can lead to health problems in newborns over time. Survival rates for these calves are low. BC contains numerous bioactive molecules, including immunoglobulins (IgG, IgA, IgM, IgE, IgD), growth factors, hormones, lysozyme, lactoferrin (LF), and lactoperoxidase. These components make colostrum an extremely important nutrient [2]. BC has a wide range of immune effects on health, which are considered to be mediated by the bioactive components of BC. Following BC administration, changes occur in the gut microbiota, and antimicrobial and immune effects are mediated through LF. Thus, BC has significant potential in preventing infections. Based on the gut microbiota effects of BC, it is hypothesized that modulation of the tumor microenvironment in colorectal cancer (CRC) may also influence metastasis [3]. Animal milk and dairy products can be used in cancer treatment. The proteins in milk are shown to be valuable sources of various biological activities, including immune modulation, cholesterol reduction, antihypertensive, antimicrobial, and anticancer activities, in preclinical studies. Moreover, LF, which is found in high amounts in mammalian milk, has been reported to decrease non-small cell lung carcinoma [4]. Anticancer or cytotoxic actions on cells deriving from multiple tumor sources are also found in various caseins found in milk, such as α-lactalbumin, bovine α-lactalbumin with cytotoxic properties against tumor cells, β-lactoglobulin, and bovine serum albumin, as well as other whey proteins; conjugated linoleic acid, the membrane surrounding milk fat globules, butyrate, and several milk-derived lipids; and other protein components, such as calcium, LF, lactoferricin (Lfcin), and casomorphins [5].

The immune effects of colostrum are well known, but the effects of exosomes isolated from BC (BCE) on human health are not completely understood. Small extracellular vesicles (sEVs) surrounded by a membrane (EVs) are released from cells to promote communication between cells. Exosomes are the smallest vesicles in the EV family and have a size of approximately 30–150 nm [6]. sEVs provide certain advantages in terms of drug delivery potential, such as their biocompatibility, low cost, and high availability. Additionally, colostrum provides a higher exosome yield than mature milk [7]. sEVs are among the most intriguing structures that can play a role in drug delivery. sEVs carry their own native proteins that help cells take up the vesicles and their cargo. Through their biogenesis processes and natural functions, which enable the transport of materials like proteins and nucleic acids from one cell to another, exosomes are natural structures capable of crossing the cell membrane. Due to their low immunogenicity, their systemic circulation increases. With these characteristics, sEVs are thought to be able to enhance drug delivery capacity, especially when treating systemic conditions, including cancer. For this reason, sEVs stand out as natural molecules that are extremely suitable for advanced bioengineering studies [8]. Current research comparing the anticancer effects of milk exosomes obtained during early, mid-, and late lactation periods with colostrum is quite limited [9]. Milk exosomes may be collected from various sources, including, humans, cows, buffaloes, camels, goats, sheep, yaks, and pigs [6]. Another reason for the preference for milk exosomes is their scalability and cost-effectiveness. It has been reported that 1 mL of milk contains 10^12^ extracellular vesicles of BME size [10]. Various in vivo and in vitro studies have demonstrated that BMEs have therapeutic effects in different disease models. Their exosome content primarily exhibits anticancer effects, and milk-derived exosomes are frequently highlighted as promising carriers for transporting chemotherapy agents, such as celastrol, in lung cancer cases. As noted in one study, exosomes obtained from colostrum produced during different lactation stages were found to contain a significant number of miRNAs that play a role in immune activity [6].

Cancer is a serious health problem that calls for quick diagnosis and medical action. According to estimates, about 27.5 million new cancer diagnoses are expected to emerge each year by the time we reach 2040. These estimates represent a 61.7% rise relative to the current data [11]. Cancer is a disease with highly complex mechanisms that develops as a result of the accumulation of epigenetic and certain genetic alterations that interfere with the normal cycle of cell growth and death. In this disease, mutations that activate proto-oncogenes, together with the loss of function in tumor-suppressor genes, disrupt normal controls and cause cells to proliferate uncontrollably [12]. There are a number of epigenetic changes that support the formation of the tumor microenvironment, such as DNA methylation and histone modification. These epigenetic changes protect the tumor microenvironment from the immune system [13]. Furthermore, effects that cause inflammation and infection, such as cytokine release, support tumor growth [14]. Current cancer treatments are highly toxic and lack sufficient selectivity. This has led to the search for natural and new approaches to cancer prevention and treatment [15]. In this context, exosomes stand out, with a number of advantages. One example of these advantages is the lipid bilayer structure of sEVs. The bilayer structure helps sEVs protect their contents from enzyme action. This bilayer structure allows molecules to be transported intact to the target site [16]. When bevacizumab was delivered in exosomes, its effects lasted twice as long. This demonstrates that exosomes reduce toxicity, increase bioavailability, and are selective [17]. Exosomes possess advanced targeting capabilities thanks to the natural proteins and ligands found on their surface. This advanced targeting has become important in alternative therapies developed to prevent tumors [18]. Moreover, sEVs can remain stable in circulation for extended periods due to their natural origin. This review article addresses the therapeutic effects of BCEs and BME in cancer treatment and is supported by clinical trial findings. The biological contents and fundamental components of exosomes are analyzed, and their mechanisms of action in cancer treatment are explained. Human milk exosomes are described in this review due to their physiological importance and active role in immune development [19]. However, they are not directly equivalent to animal milk exosomes in terms of molecular content and function. Therefore, while this review focuses primarily on animal milk-derived exosomes, human milk exosomes provide a framework for physiological comparison and interpretation [20]. Moreover, although some similarities have been observed, exosomes isolated from the milk of different species are not directly interchangeable in terms of biological effects. This review focuses on the preclinical level and adopts a critical approach that includes not only positive effects but also potential negative effects. The anticancer effects of milk- and colostrum-derived exosomes, as well as the molecular and cellular mechanisms underlying these effects, are discussed. The potential limitations, context-dependent adverse effects, and biological uncertainties that may be encountered in the clinical application of milk-derived exosomes are also addressed. This review provides a mechanism-based and balanced framework that facilitates the realistic interpretation of findings from preclinical studies and their examination in translational applications.

## 2. Bovine Milk and Colostrum Exosomes

### 2.1. The Structure and Basic Components of Exosomes

Exosomes have a wide and heterogeneous molecular range. Exosomes found in many populations share certain structurally conserved features [21]. Furthermore, exosomes are structures formed in the endosomes of eukaryotic cells and included as part of the EV group. They have a double-membrane structure measuring 30–150 nm [22]. Exosomes are found in a spherical form in solution, but after artificial drying processes are applied during the preparation process, they can have a biconcave or cup shape [23].

As they derive from endosomal structures, EVs carry a variety of protein families that play a role in intraluminal vesicle (ILV) formation. Among these proteins are CD9, CD63, CD81, TSG101, and Alix, from the tetraspanin family [24,25]. Although CD9 was the first tetraspanin identified in dendritic cells, CD63 and CD81 are highly expressed in many types of exosomes. Due to this high expression, these proteins stand out as important markers for the detection of exosomes [21]. Exosomes contain high numbers of proteins. Proteins are associated with membrane fusion, cell-to-cell signals, and protein movements. Proteins can influence growth by affecting the tumor microenvironment. SYT7 proteins found in exosomes stimulate tumor angiogenesis, supporting the uptake of nutrients and oxygen, which in turn accelerate metastasis [26]. Additionally, annexins, Rab proteins, and flotillins; major histocompatibility complex proteins (MHC I and MHC II); heat shock proteins (HSP70, HSP90, Hsc70); and cytoskeletal proteins such as actin, myosin, and tubulin are also commonly found in exosomes. [24,25]. HSPs take part in protein-folding and the transport of proteins. Besides regulating cellular stress responses and antigen presentation, Rab proteins found in exosomes play a role in the formation and release of exosomes. These proteins are abundant in exosomes, along with Alix and TSG101, which are multivesicular body-formation proteins [21]. Exosomes from human dendritic cells and enterocyte contain enzymatic proteins, including pyruvate kinases and peroxidases [27]. Proteins associated with the ESCRT complex are important for generating MVBs and packaging ILVs [25]. In addition to extracellular matrix (ECM) remodeling, matrix metalloproteinases (MMPs) regulate intracellular signaling and cell–cell communication through the proteasomal processing of exosomal cargo [27]. Moreover, certain proteins found in exosomes, including TTN-AS1 and Rab27b, can directly influence tumor development by promoting the growth and spread of cancer cells [26]. Various studies have shown that exosomes contain key transcription factors. These factors are linked to the Notch, Wnt, and Hedgehog signaling pathways [21]. Furthermore, some proteins in exosomes have negative effects, such as making the immune response less active and enabling tumors to escape the immune system. Many tumor-associated proteins were identified in exosomes in studies. Some of these identified proteins were confirmed to be target molecules in cancer treatment [26]. Significant changes in protein composition can be detected in exosomes released from cancer cells. As an example of these changes, the continuously active oncogenic variant EGFR vIII was reported in glioblastoma-derived exosomes. This demonstrates that molecules promoting invasion are enriched in the exosomal load and reveals the dynamic nature of protein transport [28]. The proteins in exosomes may not be evenly distributed across all subpopulations. This demonstrates that structurally similar vesicles with different contents can be released from the same cells [21].

Lipids found in exosomes consist mainly of phospholipids, cholesterol, and triglycerides, and take part in the mechanisms that lead to tumor development and progression [26]. Moreover, exosomes are rich in lipid components such as phosphatidylcholine, phosphatidylethanolamine, phosphatidylinositol, phosphatidylserine, sphingomyelin, glycosphingolipids, and ceramides [22,24]. Phospholipids found in exosomes can alter the structure of cell membranes. This effect can regulate signal transduction and membrane permeability. This can be part of the development of the tumor microenvironment [26]. The membranes of exosomes are rich in phosphatidylserine and cholesterol [27]. By increasing the rigidity of the membrane and its resistance to degradation, the transport of nucleic acids and proteins is facilitated [25]. The lipids present in the exosomal membrane can vary depending on various factors. The lipid composition of exosomes can be altered by signals coming from the environment. Culturing PC3 cells with hexadecylglycerol resulted in the release of exosomes loaded with ether lipids. This resulted in changes in the protein profile. The distribution of the double-membrane structure in the exosome membrane is asymmetrical; sphingomyelin is usually found on the outer surface, and phosphatidylserine found on the inner surface. In apoptotic and malignant cells, phosphatidylserine is transported to the outer layer and sends an “eat me” signal to macrophages [29]. High sphingomyelin and phosphatidylinositol levels help exosomes remain stable in body fluids and across varying pH levels [22]. Enteropathogenic bacterial secretory particles are derived from the intestinal mucosa. These particles can promote the production of nanoparticles that are similar to exosomes, characterized by elevated sphingosine-1-phosphate, CCL20, and prostaglandin E2 contents. These molecules facilitate the advancement of colon cancer by supporting the attraction and expansion of Th17 cells [26]. Additionally, the asymmetric exosome’s stability is supported by the lipid arrangement of internal and external membranes [25]. Furthermore, the exosome membrane includes lipid rafts. These domains contain tyrosine kinase, Src, and several proteins [22]. Phospholipids participate in signaling pathways that control apoptosis and autophagy. These pathways can contribute to the expansion of tumors and their spread. The nutrients and energy needed for cell growth are provided by triglycerides found in exosomes. In contrast, the metabolic pathways involved in triglyceride synthesis are highly active in tumor cells. This is due to differences in the growth of cells and their potential to spread [26]. Different lipid compositions can be used as potential markers to check the purity level of isolated exosomes. Studies have shown that the lipid content of small EVs differs from that of large EVs isolated from lung cancer patients. This observation shows that more sensitive biomarkers could be developed for specific types of EVs [29]. The nucleic acid content of exosomes includes mRNA and microRNA (miRNA), as well as long non-coding RNA (lncRNA), circular RNA (circRNA), ribosomal RNA (rRNA), transfer RNA (tRNA), small nucleolar RNA (snoRNA), small nuclear RNA (snRNA), and piwi-interacting RNA (piRNA) [24]. lncRNAs have regulatory functions such as cellular differentiation and cell cycle regulation. circRNAs function as competing inhibitors against microRNAs in the control of protein activities [27]. miRNAs contribute to cell-to-cell communication in normal and pathological conditions. In CRC, miR-29a levels are elevated. MiR-29a is delivered from cancer cells to endothelial cells. Increased vascular permeability may occur with these miRNAs. CRC progression and metastasis are promoted. This shows that miR-29a has potential as a biomarker and therapeutic tool in CRC [26]. From these features, the health of the cell and how well it is functioning can be understood [25]. Exosomes may play a role in disease diagnosis. Also, exosomes have non-invasive potential for disease prognosis. These properties have been investigated in recent studies [23]. DNA and RNA can be transported by exosomes. Because exosomes lack histones and double-stranded structures, they do not carry nuclear genomic DNA. This must be considered to understand the impact of exosomes on disease progression and immune responses [28]. These properties suggest that miRNAs can be used as biomarkers in cancer. miR-125b-3p, miR-122-5p, and miR-205-5p were increased in plasma exosomes in pancreatic cancer. This shows the potential of exosomes as non-invasive biomarkers. Tumor cell proliferation can be altered by exosomal miRNAs. Exosomes, which are molecules that can be transferred into cells, may contribute to this phenomenon. The Wnt signaling pathway may be affected by an exosomal miRNA, leading to an increase in miR-150. Tumor cell proliferation may be affected by exosomes, as shown in this example [26]. Exosomal miRNAs may be involved in the processes of exocytosis, hematopoiesis, and angiogenesis [23]. miRNAs can be selectively packaged into exosomes through various mechanisms and specifically transported to the target region. Sequences called “EXO motifs” play a critical role in packaging. These motifs are found in exosomal miRNAs. RNA transport proteins such as β-box protein 1 (miR-144 and miR-233), annexin A2 (viral RNA), hnRNPA2B1 (miR-198 and GGCU motif RNA), and SYNCRIP (EXO motif RNA) play a role in this process [28].

### 2.2. Distinctiveness of Exosomes Isolated from Milk and Colostrum

Breast milk is a biological fluid that yields large amounts of exosomes and, along with its regenerative properties, its effects have been known for a long time. Milk exosomes demonstrate strong therapeutic potential owing to their tolerance of different species and their ability to induce immune or inflammatory responses. However, existing isolation protocols are limited due to the presence of unwanted components such as fat and casein. The fats in milk can bind to exosomes and thus alter their physical properties and functions [30]. BM provides a range of bioactive substances that play key nutritional and immune-related roles in both newborns and humans. Milk and colostrum exosomes from cows with high immune responses induced greater metabolic activity in Caco-2 cells than those from low-response cows. While colostrum exosomes showed a tendency toward increased caspase-3 activation, this effect was not observed in milk exosomes. Additionally, cell viability was higher in milk exosomes compared to colostrum exosomes after 24 and 72 h of incubation. To facilitate comparison, key conserved miRNAs and lactation stage-dependent differences in milk-derived extracellular vesicles across species are summarized, as shown in Table 1 [31]. Exosomes found in mature milk are rich in proteins linked to molecular transport and programmed cell death. In contrast, colostrum exosomes contain more proteins associated with immune and inflammatory processes, antimicrobial peptide activity, cellular growth, and complement system activation. Functional enrichment analyses show that exosomes from colostrum contain higher levels of immune- and growth-related proteins than those from milk [32]. Colostrum carries more EVs, at higher concentrations, than mature milk. Exosomes obtained from colostrum reduce the activation of apoptotic pathways (Bax, p63, caspase-3) and proinflammatory genes (TNFα, IL6, IL1β). Mature milk EVs are not effective regarding apoptosis gene expression but reduce TNFα and IL6 expression [33]. EVs derived from colostrum and mature milk increased the expression of genes supporting intestinal barrier function (TJP1, CLDN1, OCLN, CDX2, MUC2, IGF1R) and reduced LPS-induced inflammation and apoptosis [34]. Milk contains miRNA, which is essential for regulating the immune system and supporting its development, and is highly conserved across species. miRNAs present in exosomes isolated from both colostrum and mature milk are similarly present in the milk of humans, cows, and goats. Immunity-related miRNAs (miR-30a-5p, miR-22-3p, miR-26a) are observed in all three species [35]. Quantitative proteomic analyses have shown that the protein content of exosomes varies across different stages of lactation. Proteins associated with immune control and growth are present in much higher amounts in colostrum exosomes [36].

### 2.3. A Brief Overview of Isolation and Characterization Methods

Ultracentrifugation (UC) is among the most common techniques used to isolate exosomes. In ultracentrifugation (UC), dead, aggregated, and apoptotic particles are removed through sequential high-speed spins, allowing for exosome isolation based on pelleting behavior. Density gradient (DG) centrifugation, a modified UC approach, is considered the reference method, enabling high-confidence isolation through differential separation [38]. Compared with commercial stacking or column-based kits, UC requires more time but results in a higher exosomal RNA yield. Therefore, UC provides an advantage for miRNA analysis [39,40].

Another isolation method is called size-exclusion chromatography (SEC). The SEC technique uses the original biological sample as the mobile phase, and a porous gel-filtration polymer forms the stationary phase. Larger proteins are eluted more rapidly by replacing them in smaller vesicles that are obtained from earlier fractions, followed by soluble proteins that are not enclosed by membranes [41]. Compared to UC, higher protein recovery and purer fractions are obtained in pure exosomes [42]. Consequently, SEC stands out as a suitable isolation method for high-integrity and high-purity applications. Polymer-based precipitation is an alternative and convenient supplement used for exosome isolation [43]. Polyethylene glycol (PEG) is the polymer used most frequently; it prevents melting and enables exosome disruption at low centrifugation speeds [44]. In addition, PEG-based precipitation is a low-durability and highly applicable exosome isolation method [45].

Ultrafiltration isolates exosomes based on how their size differs from surrounding components, using membrane filters with precise and comprehensive weight cut-off values. The effects depend on various factors, including filter and membrane type and pore size. Regenerative cellulose membranes provide the best results [46]. In ultrafiltration, two different setups are employed to separate BM. High-purity exosomes were obtained by removing impurities through 150 nm and 50 nm SiNx membranes [47]. This is one of the alternatives to UC in clinical applications and causes less physical damage to exosome samples [48].

Exosome characterization requires detailed analysis of the morphological, physical, and biochemical properties of these vesicles. Based on the MISEV criteria published by the International Society for Extracellular Vesicles (ISEV), the use of multiple complementary techniques is recommended for characterization [49]. Transmission electron microscopy (TEM) are often used to obtain very-high-resolution exosome morphology. This method can be used to determine whether exosomes are actually present. Gold-labeled antibodies can be used to identify markers on exosome surfaces. Surface protein identification is facilitated. Immuno-EM techniques are performed using these antibodies [50]. Scanning Electron Microscopy (SEM) is a technique that provides a detailed visualization of the three-dimensional structure of the surface. Cryo-EM is used to obtain the closest image to the natural state by avoiding distortion in the samples. The surface properties of exosomes can be determined using Atomic Force Microscopy. This technique provides detailed results even in liquid media [51]. Nanoparticle tracking analysis (NTA) is used to measure size in the 10–100 nm range. Through monitoring Brownian motion, subpopulations can be distinguished using fluorescence mode [52]. Dynamic Light Scattering (DLS) analyzes changes using light scattering. This analysis provides a size estimate. If the structure is complex, it may produce inconsistent results [53]. As exosomes pass through the membrane, electrical resistance fluctuations occur. These fluctuations are used to calculate size and density using Tunable Resistance Pulse Spectroscopy (TRPS) [51]. Rapid capture for miRNA analysis can be achieved with small samples. Microfluidic systems utilize this method. Flow cytometry identifies surface antigens using fluorescently labeled antibodies. Newer systems can detect smaller particles [53]. Western blot (WB) and ELISA are used to validate surface and intracellular proteins. NP-TRFIA rapidly detects tumor-associated proteins in urine and cell supernatants without requiring pretreatment [52]. SP-IRIS enables phenotypic analysis and the digital quantification of exosomes above 50 nm in size without requiring labeling [53]. Label-free methods such as nanoplasmonic sensors and surface plasmon resonance (SPR) provide high-throughput cell or particle characterization and concentration analyses [54]. Mass spectrometry (MS) provides a comprehensive profile of the proteome and lipidome [51].

As discussed earlier in Section 2.2, BME isolation may be affected by the co-isolation of abundant proteins such as caseins, which remains an important consideration when evaluating isolation strategies.

## 3. Exosomes Mechanisms of Action in Cancer Therapy

**Figure 1:**
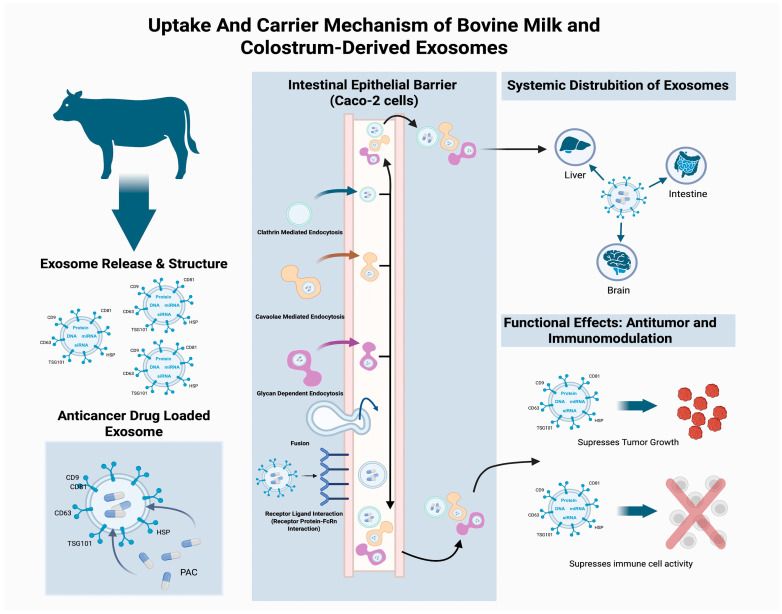
Uptake and cCarrier mMechanism of BM and BC-derived Exosomes: This figure visually summarizes how exosomes derived from BM and colostrum overcome the epithelial barrier of the intestine (Caco-2 cell model) and how they are distributed throughout the body. On the left side, the formation process and structural components of exosomes (lipids, proteins, mRNA, and miRNA) are shown under the heading “Exosome Release and Structure.” This panel also describes how anticancer therapeutic agents like paclitaxel can be incorporated into exosomes and designed as targeted delivery systems. The middle section shows how exosomes are internalized into cells by intestinal epithelial cells via clathrin, caveolae, and glycan-mediated endocytosis mechanisms, then transported within the cell via receptor–-ligand interactions and membrane fusion. The process depicted in the figure describes the transfer of exosomes into the bloodstream. The right panel demonstrates the transport of exosomes to organs and their ability to reduce tumor growth in these organs. This figure shows that exosomes can cross certain biological barriers. Furthermore, these structures can act as transport systems, delivering their cargo to various tissues [57,58]. The uptake mechanisms shown in the figure are supported by experimental evidence; however, the overall biodistribution is presented not as a direct confirmation of a single experimental pathway, but as a schematic synthesis of findings from preclinical studies. Preclinical studies have detected the accumulation of milk-derived exosomes in the brain under specific experimental conditions (in mouse models subjected to oral gavage). Therefore, the ability of exosomes to cross the blood–-brain barrier should not be interpreted as meaning they cross homogeneously under all conditions [59].

Milk-derived exosomes have shown potential as anticancer activity and drug delivery systems (Figure 1) in preclinical cancer models. In vivo and in vitro, they have demonstrated tumor-suppressive effects such as apoptosis induction and inhibition of tumor growth. Additionally, both of these biological roles may be exhibited by milk exosomes depending on the context. Some studies have indicated that oral administration may have tumor-promoting effects [55]. Considering this dual effect, the experimental context, exosome source, transported cargo, and dose should be carefully evaluated when interpreting the model system [56].

### 3.1. Uptake and Carrier Potential

Exosomes are very small exogenous vesicles that can be secreted by many cells in the body. These vesicles function to transmit signals to each other. They contain biological substances such as proteins, lipids, and DNA and RNA. They can transport these substances to target cells [60]. Exosomes can cause phenotypic changes by transferring the molecules they carry to recipient cells using fusion mechanisms. Through the circulatory system, they can reach tissues distant from the cells where they are secreted and bind to their receptors (as shown in Figure 1). Exosomes are attracting attention due to their increased heterogeneity in pathological conditions such as cancer [57]. Exosomes can both promote and inhibit tumor growth during cancer progression. Exosomes, which have a diameter of approximately 30–150 nm, play a role in shaping the extracellular matrix (ECM) [61]. These observations are presented as mechanistic examples, illustrating context-dependent exosome functions rather than disease-specific therapeutic outcomes. Studies show that exosome levels in individuals with blood cancer are about double those found in healthy controls, indicating that diseased organs produce higher amounts of exosomes [57]. Multiple studies provide mechanistic illustrations of the exosome-mediated signaling pathways within the tumor microenvironment. Additionally, research has revealed that exosomes secreted by cancer cells modify stromal cells, leading to increased tumor growth and invasion, and also stimulate VEGF signaling, acting in an autocrine manner in endothelial cells to support tumor angiogenesis. Furthermore, through the movement of PD-L1, TGF-β, and other immune dampener factors, exosomes contribute to reduced tumor immunity. Research has shown that exosomes released by dendritic cells and tumor cells can activate CD8+ T cell-mediated antitumor responses by expressing molecules like MHC class I and various HSPs [61]. Because they are highly stable and biocompatible, exosomes are gaining attention as both anticancer drugs and gene-based delivery approaches applied to experimental preclinical cancer models. These properties enable exosomes to contribute an important mechanistic function in stimulating anticancer immune responses and transporting therapeutic agents [60]. When explaining the ways in which exosomes act on cancer cells, the uptake and carrier potential of exosomes should first be discussed. Exosome biogenesis begins with plasma membrane invagination, during which the ESCRT complex sorts cargo into intraluminal vesicles and drives multivesicular body (MVB) formation. After exosomes fuse with the plasma membrane, they are released into the extracellular environment. Proteins such as CD, CD63, and CD81 are involved in cargo sorting (as shown in Figure 1) [58]. Endocytosis, fusion, and receptor–ligand interactions are active in cellular uptake. For example, Caco-2 cells have been shown to have a high internalization capacity; inhibitors of NaN3, clathrin, caveolae, and microinoculation suppress this process [62]. Additionally, fluorescent labeling studies have confirmed that colostrum and milk exosomes are taken up by endocytosis [31]. One of the points to consider when discussing the uptake and carrier potential of exosomes is oral administration and gastrointestinal barrier crossing. As a mechanistic basis for oral delivery, several studies have demonstrated that exosomes obtained from milk and colostrum remain stable in the gastrointestinal environment and can cross the epithelial barrier. It has been reported that BMEs are permeable through Caco-2 cell layers via an FcRn-dependent mechanism, with uptake being approximately threefold higher in differentiated cells, underscoring the importance of epithelial maturation. However, in vivo biodistribution may vary depending on physiological and tissue-specific factors, limiting direct extrapolation in vitro models [63]. BMEs mediate apical-to-basolateral miRNA transport and are internalized via glycan-dependent endocytosis and macrophage scavenger receptors [64]. Additionally, in vivo experiments indicate that miRNAs obtained from milk can be taken up by cells of the digestive tract after they build up in the intestinal accumulation, liver, spleen, and brain. These findings provide mechanistic insight into systemic uptake rather than direct evidence of therapeutic efficacy [65]. The lipid bilayer structure and surface proteins of exosomes facilitate endocytosis, providing an advantage in the transport of drugs and nucleic acids. Exosomes derived from cow milk have served as carriers for drug delivery through loading them with small molecules, siRNA, and miRNA; it has also been shown that PAC-loaded exosomes suppress tumor xenografts [7]. PS-modified AExos increased cellular internalization and led to a threefold increase in antibacterial activity, showing that exosomes could strongly support drug concentration in infection sites [66]. In the tumor microenvironment, exosomes regulate immune responses through immunosuppressive ligands and serve as potential therapeutic carriers [67,68].

### 3.2. Apoptosis Induction

The anticancer effects of colostrum and milk exosomes, particularly those derived through the induction of apoptosis via their miRNA content, are highlighted as a key mechanism. Upregulation of miR-148a, miR-15b, and especially miR-27b has been demonstrated in exosomes derived from buffalo milk. miR-27b mimicry induces apoptotic death in CRC colorectal cancer cells by activating caspase-3, disrupting mitochondrial membrane potential, and triggering endoplasmic reticulum stress (Table 2) [69]. LF exosomes regulate apoptosis at the gene level by downregulating Bcl-2 and upregulating Bid, leading to delayed apoptosis and selective cytotoxicity in breast cancer cells [70]. Protein contents also play a critical role in apoptosis regulation. Camel milk exosomes elevate the expression of Bax and caspase-3 but suppress Bcl-2 in HepaRG liver cancer cells, accelerating apoptotic cell death by intensifying DNA damage. These effects were particularly pronounced in colostrum-derived exosomes. Moreover, due to their comparable protein composition, BMEs are likely to possess similar apoptosis-inducing potential. However, species-specific differences in exosomal cargo may influence their biological effects (Table 2) [9]. The alpha-lactalbumin-derived HAMLET complex induces apoptosis in glioblastoma and bladder cancer models, and milk exosomes have been shown to trigger antiproliferative and apoptotic pathways in various cancers [71]. Milk exosomes loaded with curcumin or resveratrol induce p53-independent cell cycle arrest and mitochondrial apoptosis at low doses by entering cells through clathrin-mediated endocytosis and overcoming ABC transporter barriers [72]. Milk-derived exosomes from various species act as delivery platforms in preclinical cancer models, with goat milk exosomes inducing miR-34a-dependent apoptosis and horse milk exosomes, enhancing apoptosis via anticancer peptides [73]. Additionally, the regulatory functions of exosomes obtained from milk on the immune and intestinal systems in the context of their effects on apoptosis are also noteworthy. miRNAs such as miR-4334, miR-219, and miR-338 play a role in reducing intestinal inflammation and apoptosis, as demonstrated in a study using exosomes derived from porcine milk [74]. Colostrum-derived exosomes exhibit greater suppression of apoptosis-related genes than mature milk exosomes, although both reduce LPS-induced apoptosis via downregulation of intestinal barrier genes [33]. Collectively, these data suggest that milk exosomes exert context-dependent regulatory roles, both promoting apoptosis in tumor cells and suppressing apoptosis under inflammatory conditions. 

### 3.3. Modulation of Cancer-Related Inflammation

Exosomes derived from milk and colostrum can affect the pathways that cause inflammation. They can achieve this effect through miRNA, TGF-β, and other immune-regulating proteins. In LPS-stimulated systems, exosomes produced from milk have been shown to increase cell survival by reducing NF-κB and PI3K/AKT activation. This reduction lowers the IL-6 and TNF-α release [75]. A similar effect was observed with porcine milk-derived exosomes. LPS-induced inflammation was also reduced by porcine milk exosomes. Given their similar bioactive content, BMEs may have a similar effect to that found in porcine milk through inhibiting the p53/FAS/Caspase-3 axis. The inhibition of this signaling pathway results in reduced cell death. Exosomal miRNAs such as miR-4334, miR-219, and miR-338 may exert these collective regulatory effects (Table 2) [74]. Exosomes from breast milk can contribute to immune regulation. Exosome molecules are resistant to digestive enzymes. This property allows exosomes to transport TGF-β and miRNAs to their targets. This suggests that exosomes participate in immune regulation and inflammatory responses. Oxidative stress suppression and increased proliferation in the intestinal epithelium have been identified as effects of exosomes. Exosomes also reduce inflammation. These effects have been demonstrated in various animal models. In addition to their protective effects, exosomes may also carry potential risks due to certain mechanisms. In some conditions, TGF-β isolated from exosomes stimulates epithelial mechanisms. This makes tumor cells more aggressive in the environment [71]. Another type of exosome with inflammatory effects is exosomes derived from camel milk. Moreover, camel milk exosomes increase reactive oxygen species (ROS). This increase triggers stress responses in cancer cells. Camel milk exosomes show antiproliferative and antimigration effects in CRC cells via the suppression of TNF-α and IL-6 expression [76]. Therefore, apoptosis and inflammatory effects are promoted by exosomes from camel milk. These effects can be exerted in cancer cells through gene expression-level regulation. BM and camel milk have similar molecular contents. It can be assumed that BMEs may also exhibit similar effects in cancer cells because of their similar molecular content. However, species-specific differences and limited comparative data warrant cautious interpretation [77]. Exosomes derived from cow milk suppress inflammation in murine models of ulcerative colitis. Therefore, treated mice showed increased intestinal barrier gene expression in an in vivo study. These genes include TJP1, CLDN1, and OCLN [78]. Intestinal damage is reduced by milk exosomes. Milk exosomes lower the expression levels of the genes that cause inflammation in necrotizing enterocolitis (NEC) models [79]. Inflammatory processes can be targeted when exosomes are used as drug carriers [80]. Pure milk exosomes cannot induce proinflammatory responses if they are administered intravenously. Moreover, the suppression of anti-inflammatory cytokines can be observed when pure milk exosomes are loaded with miRNA. In inflammation-related tumor microenvironments, such as CRC, as mentioned earlier in this review, the suppression of NF-κB and PI3K/AKT signaling and pro-inflammatory cytokines by milk-derived exosomes may limit tumor-supportive inflammation (Table 2) [81]. BMEs and BCEs show general suppressive effects in inflammatory cytokines in tumor microenvironments through several immune regulators. The suppression of cancer progression occurs through the reduction in intestinal and systemic inflammation caused by exosomes. Understanding the mechanisms through which these molecules regulate inflammation is important for further cancer prevention and treatment studies using exosomes.

### 3.4. Immunomodulatory Effects

Exosomes exhibit duality in their immune-regulator effects, showing both immunostimulatory and immunosuppressive effects. The differentiation of CD4+ T cells suppresses antitumor responses. Antitumor suppression occurs due to the inhibition of PTEN expression by exosomal miR-214 and IL-10 production increases, creating a tolerogenic environment [82]. Fas/FasL interaction suppresses TCR signaling, inducing apoptosis in CD8+ T cells (Table 2) [83]. In addition, exosomes that carry TGF-β1 and PGE2 induce MDSC differentiation. Antigen presentation and immunosuppressive activities increase via this differentiation. Tumor-supporting phenotype maintenance is not affected by these signals, enhancing immune suppression [82]. Exosomes also exhibit immunostimulatory effects. HSP70-bearing exosomes play a role in reducing the migration and cytolytic activity of NK cells (Table 2). Additionally, exosomes released from tumor microenvironments and exposed to heat stress support dendritic cell maturation by increasing CTL activation. Dendritic cell-derived exosomes (Dex) have been shown to enhance antigen presentation as they contain molecules such as MHC-I/II and CD86. Dex administration reduced CTL activation in mouse models. NK cell activation and enhanced T cell responses have been observed in clinical studies [83]. In addition, plant exosomes have been shown to regulate cytokine production in colorectal cancer. This suggests that exosomes enhance immune responses [84]. Colostrum- and milk-derived exosomes play a crucial role in immune regulation. A large portion of the miRNAs in milk exosomes are associated with immune responses. These miRNAs play a role in regulating B cell development, IgM synthesis, T cell activation, and Th1/Th2/Th17 responses [85]. Proteins that show microbial growth inhibition and homeostasis-supporting effects are abundant in colostrum [36]. Moreover, exosomes obtained from goat milk show suppressive effects in inflammatory processes. This suppression occurs via reductions in IL-18 and MMP9 gene expression. An increase in MUC2 and IL-8 levels supports mucosal immunity. This increase also occurs when using goat milk exosomes [86]. Antigen presentation is modulated by factors such as immunoglobulins and TGF-β, which are abundant in BME and BCE. The modulation of the immune activities of BMEs and BCEs is associated with these factors, as well as tumor-supporting effects. TGF-β2 elevation creates an invasive phenotype in breast cancer cells; this is an example of the tumor-supporting effects of the regulatory factors of BCEs and BMEs, along with their positive effects on immune responses [85]. Mature milk is less effective than colostrum in suppressing apoptosis and inflammatory genes, as shown by an analysis of 329 miRNAs from exosomes obtained from colostrum and mature milk [87]. The suppression and stimulation of immune responses was observed on exosomes in some studies. These observations reveal the duality of the exosomes’ immune modulation actions. The induction of Treg, the apoptosis of CD8+ T cells, and MDSC activation are examples of the immune-suppressive effects of exosomes. NK cell activation mediated by HSP70, dendritic cell maturation, and antigen presentation are examples of the immunostimulatory properties of exosomes. In cancer cells, a reduction in immune escape and enhancement of immune responses can be observed in molecules contained in milk and colostrum exosomes. Immunoglobulin, TGF-β, and immune-related miRNAs are present in these molecules. Studies conducted with exosomes suggest they can generally be considered safe molecules in terms of immunotoxicity [88]. A reduction in CRC cell proliferation through gene expression modulation using camel milk exosomes was also observed in one study [76]. Exosomes obtained from buffalo milk likely exhibit similar effects to camel milk. The miRNAs in buffalo milk exosomes play a role in regulating immune responses [89]. These common molecules, which are also found in BMEs, could present BMEs as a potential alternative to buffalo and camel milk. However, species-specific variations may affect their functional outcomes.

### 3.5. Exosomal Cargo with Anticancer Potential

In cancer biology, exosomes exhibit inhibitory and promoter mechanisms. These mechanisms occur through miRNAs and proteins contained in exosomes. The potential anticancer effects of BMEs are emphasized by these effects. The biological effects of exosomal miRNAs are context-dependent; miRNA effects in different studies are not identical. Different experimental conditions can influence the variability of study results regarding miRNA effects. Unless otherwise specified, the anticancer-related effects discussed in this section are derived from preclinical studies, primarily in vitro or animal models. The following studies are presented as mechanistic examples illustrating the biological roles of exosomal cargo, rather than as cancer-specific therapeutic applications. For example, miR-148a shows inhibitory effects in many tumor types. Furthermore, the engineering of therapeutic molecules can elevate the anticancer effects of BMEs [90]. The suppression of AKR1C1, AKR1C2, and CYP3A5 gene expression also causes anticancer effects. BMEs caused an increase in miR-148a-3p in cancer cell lines, and this increase suppressed the aforementioned gene expression (Table 2) [91]. Milk-derived exosomes have obtained similar effects by suppressing NF-κB signaling via miR-148a. This suppression protects the intestinal barrier, which is important in the treatment of cancer and inflammatory diseases [92]. miR-34a, found in exosomes, activates the p53 signaling pathway and promotes apoptosis. This miRNA can also suppress proliferation in various cancer types. The literature suggests that the miR-34a family are crucial regulators in the cell cycle and aging processes; therefore, their transport in exosomes offers a particular advantage in terms of antitumor activity [93]. miRNAs found in milk and colostrum exosomes can affect B cell activation, immune response, and lymphogenesis. Based on this, BMEs may support epigenetic abnormalities in large B cell lymphoma [94].

miRNAs carried by milk and colostrum exosomes can activate mechanisms that cause negative effects in addition to their positive effects [94]. Camel milk exosomes have been shown to regulate metastasis by carrying molecules derived from IGF, TGF-β, and casein. Because their molecular composition is similar to that of camel milk exosomes, it can be suggested that BMEs may also exhibit similar regulatory effects, depending on species-specific variations in exosomal cargo. Camel milk exosomes, which exhibit anticancer and immunomodulatory effects, stand out due to the effects of their LF and κ-casein mRNA content (Table 2). The regulation of ROS and oxidative stress responses occurs through the combined action of these two proteins. Increased ROS triggers apoptosis in cancer cells [95]. In addition to their natural cargo, the therapeutics loaded into exosomes through bioengineering also exhibit effective anticancer properties. For example, loading molecules such as curcumin, DHA, paclitaxel, and celastrol into milk exosomes has provided much higher bioavailability and tumor-suppression efficacy compared to their free forms. In addition to their natural cargo, therapeutics engineered into exosomes also exhibit potent anticancer effects [96,97,98]. ExoPAC and FA-ExoPAC, formulated using exosomes obtained from colostrum, provide advantages over free PAC in both in vivo and PAC-resistant cells, and can therefore be cited as examples of exosome products capable of exhibiting strongly inhibitory effects [7]. Similarly, the pH- and NIR-responsive Exo@Dox–EPT1 system developed for OSCC treatment caused significant tumor shrinkage through a combination of controlled drug release in the tumor microenvironment and photodynamic effects (Table 2) [99]. Furthermore, milk exosomes carrying miRNAs or siRNAs indicate they reduce cell proliferation in a dose-dependent manner and reduce DNMT1 levels in KRAS-mutant lung cancer models [91]. These findings suggest that exosomes derived from milk and colostrum can exert potent anticancer effects not only through natural mechanisms via anticancer cargos but also through engineered therapeutic cargos. Exosomes exhibit proliferative and selective protective effects in healthy cells. In cancer cells, exosomes induce apoptosis and inhibit metastasis. These effects suggest their potential as alternative platforms in preclinical settings [100].

Considering the aforementioned effects and their composition, colostrum and BME can be considered natural substances with functional potential in cancer diagnosis and treatment. Exosomes play an effective role in drug delivery to target cells. Mechanisms such as apoptosis induction, anti-inflammatory effects, and immune responses can be regulated by exosomes.

## 4. Therapeutic Applications in Cancer

**Figure 2:**
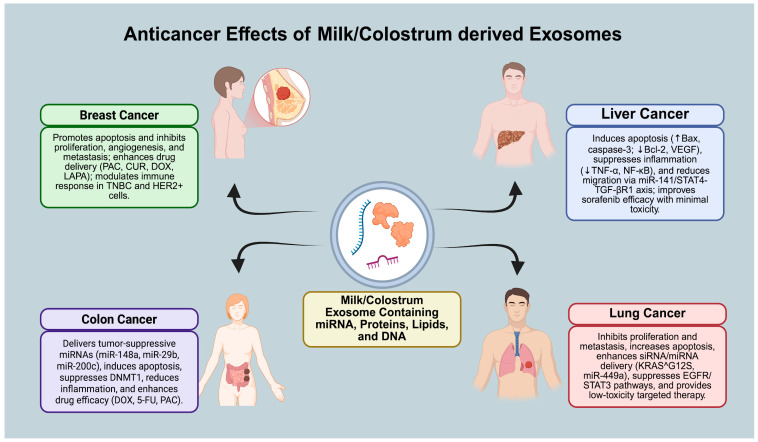
Schematic illustration of the anticancer effects of milk- and colostrum-derived exosomes on various cancer types. Exosomes carrying bioactive cargos (miRNAs, proteins, and lipids) interact with colorectal (HT-29, HCT116), liver (HepG2, HCC), breast (MCF-7, TNBC), and lung (A549, NSCLC) cancer models. Reported effects include apoptosis induction, the inhibition of proliferation and metastasis, the suppression of inflammation and angiogenesis, and enhanced drug delivery efficiency. Evidence The strength of the evidence differs by cancer type: breast and colon cancer findings are mainly supported by in vitro and 3D cell-based models, lung cancer studies include in vitro and selected in vivo mouse models, while liver cancer effects are supported by both in vitro and in vivo animal models, including efficacy and safety evaluations [103,104,105,106].

To increase the understanding of the anticancer effects of exosomes, several studies were conducted. Studies have shown that exosomes possess anticancer effects due to their bioactive components (Figure 2). Therapeutic applications of milk and colostrum exosomes in cancer have so far been evaluated primarily at the preclinical level. The studies described in this section are based on the evaluation of milk- and colostrum-derived exosomes as drug and gene delivery platforms in vitro and in animal models, and do not represent clinical treatment applications. Through the use of engineering techniques, exosomes may also exert therapeutic effects. In a study conducted using camel milk exosomes, analyses demonstrated these effects. In normal cells, exosome treatment does not cause any harm, while camel milk exosomes exhibited toxic effects on cancer cell lines. It could be said that because of their similar biological contents, BMEs may also exhibit similar effects to camel milk exosomes within a context-dependent biological framework [77]. Cancer development can also be slowed by exosomes through their antiproliferative and immunomodulatory effects. Antiproliferative and immunomodulatory activities are present in exosomes due to their rich oligosaccharide and glycoprotein contents. These oligosaccharide and glycoprotein contents are more abundant in BCEs [101]. Exosomes can carry lipophilic and hydrophilic anticancer agents. This is due to their structural stability and biocompatible membrane. This structure allows exosomes to be transported through the gastrointestinal tract without degradation, allowing them to reach their target sites [91]. In vitro studies have shown that exosomes derived from breast milk can be internalized by leukemia and colon cancer cells due to their RNA content. In an in vivo study, they directly affected cancer cell biology through the regulation of cellular proliferation by targeting DNMT1 with tumor-suppressing miRNAs such as miR-148a. Furthermore, they also demonstrated that unloaded camel milk exosomes slow breast tumor progression by elevating apoptosis-related markers and reducing the expression of inflammatory genes. As they have similar miRNA cargos and regulatory molecules, BMEs may likewise modulate tumor progression and apoptosis through comparable molecular interactions, in a context-dependent manner [98]. Studies analyzing the effects and usage methods of exosomes in this context have indicated that when exosomes isolated from milk are loaded with chemopreventive and chemotherapeutic drugs, their stability, solubility, and bioavailability show significant improvements. Thus, it has been demonstrated that exosomes exhibit stronger antiproliferative and antitumor with enhanced characteristics relative to free drugs in in vitro and in vivo studies. Significant findings emerged when paclitaxel and doxorubicin were loaded into milk exosomes, particularly regarding their controlled release at the tumor site and ability to lower systemic toxicity and enhance tumor specificity [102].

### 4.1. Colon Cancer

Worldwide, CRC stands as the third most frequently diagnosed cancer and is fourth highest in terms of cancer-related mortality. Current methods used in the prevention and treatment of this disease are limited due to the risk of damaging healthy cells [103]. In view of these risks, alternative methods, such as exosomes derived from milk and colostrum, which are natural, highly biocompatible, capable of passing through the GI barrier, and can be orally administered, are being researched [91,103]. In vitro studies have demonstrated that BMEs can be internalized by Caco-2 cells via endocytosis and are capable of delivering miRNAs such as miR-29b and miR-200c (Figure 2) [107]. miR-148a is suppressed in CRC. In vitro, the restoration of suppressed miR-148a via exosome delivery led to DNMT1 silencing, thereby limiting tumor-associated proliferative signaling (Figure 2) [71,90]. In vitro studies have shown that buffalo milk exosomes exhibit potent anticancer effects by increasing apoptosis, leading to mitochondrial ROS accumulation, and triggering ER stress via miR-27b in HCT116 and HT-29 cells [69]. Another feature of exosomes that is worth mentioning in this context is their drug delivery potential. In vitro, doxorubicin-loaded goat milk exosomes exhibited lower IC50 values in HCT116 cells, resulting in reduced cell viability. Thus, they showed a stronger anticancer effect than cow and buffalo milk exosomes [108]. In vitro studies have further shown that exosome formulations combining quercetin and 5-fluorouracil exhibit stronger antiproliferative effects and increased resistance to acidic conditions [109]. Moreover, the delivery of milk-derived exosomes with paclitaxel negatively affected tumor growth and reduced side effects [102]. Natural milk proteins, also found in exosomes, are effective against CRC and have been researched. Studies indicate that exosomes lower cytokine levels by inhibiting the LF NF-κB pathway and increase MK cell-mediated cytotoxicity [70]. An increase in anti-inflammatory mediators such as IL-10 and IL-13, a reduction in activities of IL-1, IL-6, and TNF, and the promotion of apoptotic responses can be achieved by polyvalent immunoglobulins obtained from breast milk. In colon cancer models, milk- and colostrum-derived exosomes have been reported to reduce inflammatory signaling through NF-κB inhibition and cytokine modulation, consistent with their anti-inflammatory effects (as shown in Figure 2). [11]. Consequently, in colon cancer, exosomes obtained from milk and colostrum could provide a strong biotherapeutic strategy thanks to their natural contents (miRNA, protein) and drug delivery properties [86].

### 4.2. Breast Cancer

One of the most prevalent cancers among women and a cause of notable number of cancer-related deaths is breast cancer (BC). Triple-negative breast cancer (TNBC) leads to a lack of ER, PR, and HER2 expression. For this reason, TNBC has limited treatment strategies. In addition, TNBC is an aggressive disease. Traditional chemotherapeutics generally cause serious adverse effects (hair loss, anemia, organ toxicity), creating a need for safer and more biocompatible drug delivery alternatives. Recent studies have highlighted milk- and colostrum-derived exosomes as promising candidates because they are highly biocompatible, can be produced at scale, and are capable of crossing the GI barrier. It has been reported that Paclitaxel, curcumin, siRNA, 5-FU, celastrol, and DHA, which are agents used in cancer treatments, can be successfully transported by milk and colostrum exosomes [97]. In vitro studies using MCF7 cells have shown that treatments with camel milk-derived exosomes increase apoptosis, reduce oxidative stress and inflammation, suppress angiogenesis and metastasis, and support immune cell activity (Figure 2) [110]. In vitro studies on camel milk exosomes combined with tamoxifen and hesperidin indicate that, compared to single treatments, exosomes provide stronger antiproliferative and anti-invasive effects against breast cancer and reduce side effects. Owing to their comparable bioactive and structural characteristics, BMEs could similarly increase the impact of anticancer drugs, while lowering treatment-associated toxicity, under specific experimental and biological conditions [111]. In an in vitro study, BMEs were modified with hypoxia-sensitive lipids and an iRGD peptide targeting TNBC. As a result, the targeted delivery of doxorubicin was enhanced and showed significant cytotoxic effects in 3D tumor models [112]. Similarly, folate-labeled and erastin-loaded exosomes showed a greater anticancer effect in MDA-MB-231 TNBC cells relative to untreated erastin in in vitro research [104]. Moreover, lapatinib-loaded exosomes suppressed proliferation at much lower doses and induced apoptosis in HER2+ SKBR-3 breast cancer cells compared to free lapatinib [113]. Studies on breast cancer using exosomes derived from mesenchymal stem cells (MSCs) have also yielded meaningful results. In one study, MSCs were loaded with paclitaxel and cisplatin. These loaded exosomes strongly suppressed growth in three-dimensional in vitro models of BT-474 and MDA-MB-231 cells and caused an increase in apoptosis [114]. Exosomes also show strong advantages in breast cancer treatment because of their inherent bioactive components. For example, miR-134-loaded exosomes have been reported to reduce the invasive activity observed in breast cancer cells, while miR-503 has been shown to positively affect proliferation suppression and chemotherapy sensitivity [115]. Furthermore, it has been shown that exosomal PD-L1 contributes to immune escape in TNBC cells (as shown in Figure 2) by suppressing T cell activation. Information obtained from these findings has shown that exosomes derived from milk and colostrum can modulate context-dependent immune responses in breast cancer. It has been shown that, depending on the context, they can cause either immunosuppression or immunoactivation (as shown in Figure 2.) [116]. Breast milk-derived exosomes have shown both positive and negative effects in targeting breast cancer cells in in vitro experimental design. Research has shown that exosomes containing high levels of TGF-β2 induce epithelial–mesenchymal changes in breast cancer cells of both noncancerous and cancerous origin [117]. In addition to this effect, some preclinical evidence indicates that HER2-enriched breast milk exosomes may be able to generate potent antitumor immune responses in vaccine formulations [73]. In summary, exosomes loaded with natural compounds and drugs have demonstrated potent antitumor activity against breast cancer cells in both cell-based and animal studies. Data from these studies suggest that BMEs and BCEs may be used in the treatment of breast cancer. Exosomes offer a natural alternative for delivering biomarkers and targeted drugs [118,119].

### 4.3. Liver Cancer

Liver cancer is the fourth leading cause of cancer-related deaths worldwide, with hepatocellular carcinoma (HCC) being the most common type. The lack of early diagnosis and treatment is the primary reason for this high mortality rate. The previously noted properties of exosomes suggest they could be potential agents in HCC, with both diagnostic and therapeutic uses [120,121]. In vitro studies have shown that BME-derived exosomes suppress EphA2 expression in HepG2 cells by inhibiting Bta-miR-141, thereby reducing migration and invasion (Figure 2). In vitro studies indicate that exosomes target STAT4/TGF-βR1 and suppress the HCC phenotype [122]. In vitro studies have demonstrated that colostrum-derived exosomes induce significant apoptosis in HepaRG cells (increasedBax/cleaved caspase-3, decreasedBcl-2), inflammation (decreasedTNFα, NF-κB, TGFβ1, Cox2), and angiogenesis (decreasedVEGF), while showing a relatively safe profile in normal hepatocytes (THLE-2) under experimental conditions. These properties are associated with high LF/kappa-casein molecule contents in colostrum-derived exosomes [9]. When exosomes released from HCC cells were examined in an in vitro study, it was determined that they enriched glycolysis/gluconeogenesis and pentose phosphate pathway proteins in a manner consistent with their migration capacity. Hypoxia-related markers such as CAV1, GADH, and CAPN1 increase their mobility in exosomes [123]. Exosomes possess the ability to deliver targeted hepatic therapy with small molecules in liver cancer. For example, in vitro analyses of exosomes have shown that shikonin loaded onto milk-derived exosomes is more effective at shortening cell lifespan in HepG2 cells compared to free shikonin. These loaded exosomes enhance delayed apoptosis while strengthening the pro-apoptotic balance via Baxincreased/Bcl-2decreased [80]. In vivo preclinical studies have demonstrated that Exo-coated mPEG-PLGA/sorafenib hybrid nanoparticles (Exo-Sora-NP) reduce systemic side effects while increasing tumor accumulation and penetration and decreasing tumor volume [124]. HTTP-Exo-M1-8, modified using surface engineering, selectively targets HepG2, causing viability to drop to 45% within 48 h. This reduces in vivo tumor volume to ~270 mm^3^ while inhibiting lysosome–autophagosome fusion. Moreover, no toxicity was observed in healthy tissue [125]. Milk exosomes are stable under low-pH conditions and can carry hydrophobic/hydrophilic drugs. Thus far, animal studies have been limited to short-term observations [126]. Exosomes can serve as a bridge in metastatic scenarios. For example, in CRC liver metastasis, the delivery of CCDC80-targeting siRNAs via patient-derived exosomes enhanced response in distant metastasis models by overcoming chemoresistance, thereby increasing survival rates after hepatectomy. The results of this study provide supporting evidence for exosome-based models in liver-targeted therapies [105]. Additionally, through combination and comparative approaches, exosomes are emerging as effective molecules in liver cancer. In the Ehrlich carcinoma model, CNP and camel milk exosomes, combined with sorafenib, reduce tumor burden and increase apoptosis (increased p53, Bax, caspase-3; decreasedBcl-2), (Figure 2) shift the MMP9/TIMP1 balance against tumor migration, suppress VEGF, and improve liver damage markers (ALT/AST/ALP decreased; albumin increased; oxidative stress decreased; Nrf2/HO-1 increased; IL-1β/TNFα decreased; histological improvement). Given their analogous molecular composition and cargo functionality, BMEs may offer comparable synergistic benefits when co-administered with conventional chemotherapeutic agents, showing variability in downstream therapeutic responses [127]. Some of the negative effects that may be encountered from consuming milk and other dairy items have shown an association with liver cancer. These effects may increase the risk of HCC through IGF-1 pathways, mTORC1 activation, and excess BCAA. Milk exosomes may synergize with HBV/HCV-associated oncogenic miRNA signatures under certain conditions; therefore, formulation, dosage range, and clinical context should be carefully determined in applications using milk exosomes [128]. Overall, milk/colostrum-derived exosomes offer a powerful biotherapeutic platform for HCC through a combination of liver biodistribution advantages, signal modulation with natural cargo, and targeted delivery via surface engineering; however, production–purification standardization and advanced phase validation are required for the transition to clinical application. [120,121,126]. Overall, these findings demonstrate that milk- and colostrum-derived exosomes modulate apoptotic, inflammatory, angiogenic, and migratory pathways in liver cancer in a context-dependent manner (as shown in Figure 2.).

### 4.4. Lung Cancer

Lung cancer, particularly non-small cell lung cancer (NSCLC), which constitutes nearly 85% of lung cancer cases, persists as a leading cause of death globally, primarily because it is typically diagnosed when already advanced [129]. In this context, exosomes stand out as biological nanocarriers with high potential in lung cancer treatment, as in other cancers, owing to their strong biological compatibility, low immune activation, and built-in homing tendencies [130]. Various studies have determined that exosomes from bovine and camel milk that carry chemotherapeutic agents and natural molecules provide potent antitumor effects. In an in vitro study, milk exosomes loaded with paclitaxel and functionalized with iRGD peptides effectively penetrated lung tumor spheroids and suppressed proliferation (Figure 2) [131]. An in vitro study indicates that curcumin-loaded camel milk exosomes have more cytotoxicity compared to the free compounds in A549 and resistant A549TR cells in a study. These exosomes further inhibit the EGFR and STAT3 signaling pathways. Due to their comparable structural stability and carrier capacity, BMEs may similarly enhance the therapeutic efficacy of bioactive compounds such as curcumin through targeted pathway inhibition, with various pathway-specific responses [96]. Similarly, exosomes loaded with curcumin or potassium hydroxystrata (KH) suppressed proliferation and increased apoptosis in an in vitro experiment [132]. The use of exosomes in tumor applications is also prominent in gene therapy. Exosomes loaded with siRNA (Figure 2) against the KRAS^G12S mutation led to significant tumor inhibition in A549 cells and mouse models in vivo [88]. Similarly, in a different in vitro study, miR-449a-loaded exosomes increased apoptosis and suppressed proliferation and metastasis [106]. In addition to these effects, the delivery of natural compounds such as celastrol via exosomes increased bioavailability and demonstrated potent antitumor effects in lung tumor cells [6]. On the other hand, as in studies on exosomes related to lung cancer, exosomes were also found to be very important in terms of their safety profiles. In an in vivo experimental model, no toxicity or morphological abnormalities were observed in major organs in mice treated with milk exosomes [133]. Furthermore, the effect of circular DNA elements and estrogen-related signaling pathways identified in dairy products on lung cancer has been investigated, and a possible link between lung cancer and these signaling pathways has been established [94]. Overall, milk- and colostrum-derived exosomes loaded with drugs, natural molecules, siRNA, or miRNA have been shown to have potent antitumor effects in lung cancer treatment. These delivery systems stand out as an important alternative option in lung cancer treatments due to their low toxicity and targeting potential [133]. Overall, these findings demonstrate that milk- and colostrum-derived exosomes inhibit proliferation and metastasis, induce apoptosis, modulate oncogenic signaling pathways, and enable low-toxicity targeted delivery in lung cancer models (as shown in Figure 2).

### 4.5. Other Emerging Cancers

Pancreatic cancer stands out as a disease associated with an exceptionally high death rate in the GI system and has a very poor prognosis. Exosomes play critical roles in metastasis, proliferation, epithelial–mesenchymal transition (EMT), and angiogenesis processes in pancreatic cancer by regulating the tumor microenvironment via the miRNAs and proteins they carry. Therefore, exosomes may serve as useful biomarkers for early detection and as carriers for therapeutic delivery in pancreatic cancer [134]. Recent preclinical in vitro and in vivo studies revealed that exosome-derived miR-125b is elevated in pancreatic cancer cells with high invasive potential and enhances cells’ ability to migrate, invade surrounding tissues, and undergo EMT. These findings indicate that miRNAs are important regulators of the aggressive phenotype in pancreatic cancer [135]. Additionally, in vitro studies have shown that milk-derived exosomes can trigger cytotoxic effects in pancreatic cancer cells while showing no effect on normal pancreatic cells. Research indicates that the elevation of Bax and caspase-3 expression is confirmed by the decrease in Bcl2 [100]. One point that should be added to this information is that the effects of exosomes in pancreatic cancer should be evaluated within their specific biological context. While some miRNAs isolated from exosomes may exhibit pro-tumor effects, some studies have shown that exosomes demonstrate potential therapeutic effects under different experimental conditions (preclinical models). This contrast demonstrates that exosomes may have a context-dependent and bidirectional role in cancer. The results of the studies reveal that their effects cannot be uniformly evaluated as anticancer or tumor-promoting, but instead depend on the molecular content and experimental environment.

Exosomes exert various effects on prostate cancer through the proteins, miRNAs, and mRNAs they contain. These effects can be classified as a decrease in tumor progression, the spread of cancer, and development of tolerance to medications. By reprogramming stromal cells, changes are created in the tumor microenvironment, thereby increasing resistance to chemotherapy. Additionally, exosomes can be used as drug-carrying biological nanocarriers due to their lipid structures [136]. Milk exosomes have some negative effects in addition to their positive effects. Continuous exposure to pasteurized milk exosomes has been reported to act as a potential risk element for many chronic diseases, including obesity, diabetes, and prostate cancer [137]. The mRNA carried by milk exosomes can produce species-specific biological effects [138]. Furthermore, milk-derived miRNAs have been found to weaken the p53–DNMT1 pathway, which is associated with prostate cancer [139].

Glioblastoma is another disease for which treatment alternatives using exosomes can be offered. Glioblastoma has a limited response to current treatments since the blood–brain barrier limits treatment penetration, and it is considered one of the most aggressive forms of brain cancer. BME and BCE stand out as natural nanocarriers in glioblastoma treatments due to their ability to cross the blood–brain barrier, their biocompatible structure, and their drug delivery potential. Chlorin e6-loaded mExo (Ce6@mExo) was developed to support photodynamic therapy; when administered orally, it crossed both the gut epithelial barrier and the blood–brain barrier, accumulated in glioblastoma tissue, and provided potent tumor inhibition upon laser irradiation [140]. Moreover, loading resveratrol into exosomes enhanced brain-targeting capacity and provided a significant antiproliferative effect in U-87MG cells [141]. Recent reviews also highlight exosomes as strong candidate platforms for drug, gene, and therapeutic molecule delivery in glioblastoma treatment [142].

## 5. Preclinical Applications of BM/Colostrum Exosomes in Cancer

Exosomes have become prominent as drug-delivery systems thanks to the biological cargoes they naturally secrete, especially in recent years [126]. BM and BC offer a scalable and economical source for exosome production, providing a biocompatible nanoplatform suitable for clinical applications [8]. Exosomes can carry many different therapeutic agents, for example, miRNA, siRNA, and chemotherapeutic drugs. They can also be functionalized to deliver drugs specifically to the target site [143]. Preclinical studies have demonstrated that milk-derived exosomes provide greater anticancer efficacy, reduced toxicity, and increased oral bioavailability compared to free drugs in cell-based and animal models. The clinical and preclinical studies summarized below demonstrate how this potential has been experimentally validated. Exosomes isolated from the milk of camels and buffalo share a comparable bioactive compositions and cargo profiles with those isolated from BM. Therefore, BM also has the potential to exert similar therapeutic effects to those reported in studies using camel and buffalo milk because of its similar contents. In support of this, research on camel and buffalo milk has been included in this section to substantiate the discussed findings. While some studies show strong effects in cancer models, others do not. This may be due to the context or experimental design

The antitumor and carrier effects of and colostrum-derived exosomes in the context of cancer have primarily been studied in in vitro and in vivo preclinical models. However, systematic searches of PubMed, ClinicalTrials.gov, and similar clinical research databases revealed no clinical studies investigating the therapeutic effects of milk- or colostrum-derived exosomes in cancer in human patients. Therefore, the current evidence remains limited to the preclinical level. Additionally, the results of the evaluated preclinical studies suggest that milk- and colostrum-derived exosomes may have promising therapeutic and translational effects on cancer cells. Well-designed human studies need to be conducted in the future to validate these effects at the clinical level.

A study evaluated the applicability of BME as a drug delivery system for TNBC by using exosome modifications with iRGD peptide, hypoxia-sensitive lipid, and neuropilin agonist to form iDHRX. In this experimental design, the degradable nature of exosomes in a glutathione-containing reducing environment facilitated release under hypoxic conditions in tumor-targeted applications. In vitro models showed a 50% reduction in cell viability following iDHRX administration (Table 3). However, despite the system’s high cytotoxicity, no negative impact on its specific tumor-targeting ability was observed. The results of this study offer an alternative approach of safe and effective drug delivery in TNBC through the application of modified exosomes. Exosomes obtained from milk have strong potential for clinical use in TNBC. Due to the hypoxia-sensitive mechanism and tumor penetration of the iDHRX formulation, clinical potential was observed [112]. One study investigated the effects of LF packaging into exosomes (exoLF) on the human BC cell line. Analyses showed that 39% of the patients exhibited late apoptosis. On cancer cell lines, the exoLF formulation can be used as a selective agent. Under normal conditions, the exoLF formulation demonstrated elevated biocompatibility. These features of the exoLF formulation may suggest that LF-loaded exosomes have potential as selective and safe molecules for targeted anticancer treatments in in vitro models [70]. In another study, aimed to enhance the anticancer efficacy of DHA, an FDA-approved agent. For this purpose, BMEs were loaded with DHA, creating a novel drug delivery strategy. Drug release profiles showed rapid release followed by controlled release. Experiments using cell-based assays indicate higher cytotoxicity and ROS production, and significantly suppressed cell migration and colony formation with free DHA compared to loaded exosomes (Table 3). This study may suggest that loading DHA into exosomes may be a more effective anticancer system [97]. In another study, developed BMEs using engineering, by loading a combination of PAC and 5-fluorouracil onto FA-functionalized BMEs. This was performed to eliminate the dose-dependent toxicity of PAC. In vitro tests showed that the FA-Exo-PAC/5-FU formulation caused a significant decrease in IC_50_ values compared to the values of either drug alone (Table 3). Increased apoptosis and increased cell migration were observed when functionalized with FA. The study data demonstrated that the FA-Exo-PAC/5-FU system enhanced anticancer activity and reduced cytotoxicity in breast cancer. As BMEs are biocompatible and can be orally administered, they were found to be suitable carriers for breast cancer treatment in this preclinical study [144]. In a study, aimed to transport miR-204 by modifying exosomes. the surface modification of bone marrow-derived exosomes with hyaluronic acid (HA) was carried out. In vitro tests showed that both HA derivatives and HA-coated mExos exhibit high cell and blood compatibility, while no systemic toxicity was observed in in vivo experiments. Mechanistically, miR-204 suppresses BCL2 and RAB22A gene expression, thereby reducing tumor growth and increasing apoptosis (Table 3). Additionally, this combination increased sensitivity to doxorubicin and reduced chemotherapy resistance. The study focused on the delivery of miR-204 with an HA coating and supported the applicability of BMEs as nanocarriers in cancer treatment in in vitro experimental conditions [145]. In another research, profiled buffalo milk exosomes and identified 21 miRNAs present at different expression levels. MiR-27b knock-down experiments revealed that this miRNA shows strong anticancer activity in HCT116 and HT-29 CRC cells and increases apoptosis, mitochondrial ROS production, and lysosomal accumulation. Evaluations showed that in highly invasive pancreatic cancer cells, miR-125b levels were markedly elevated and contributed to EMT and metastasis (Table 3). These findings indicate that buffalo milk exosomes can influence how tumor cells grow, move, and invade tissues, mainly through bioactive miRNAs like miR-27b and miR-125b. Given their comparable molecular cargo, BMEs also have the potential to exert similar regulatory effects on cancer cell behavior [69]. A study investigated BMEs as biocompatible and low-cost nanocarrier systems for drugs and gene-regulating molecules, using surface functionalization by HA to enhance tumor-targeting capacity. In vitro tests confirmed that this system increased selective doxorubicin (Dox) uptake and significantly induced apoptosis in CD44-positive cancer cells (MDA-MB-231, A549, MCF-7) (Table 3). The analyses demonstrated that the HA coating enhanced cell-specific uptake and cytotoxic efficacy. The results show that exosomes enable targeted drug delivery and increase tumor-selective responses mediated by HA-CD44 [146]. One study targeted the epidermal growth factor receptor (EGFR) to increase the availability of BMEs for drug delivery. BMEs were modified with the EGFR nanobody (7D12). The chemotherapeutic drug Dox was loaded onto BMEs and accumulated in EGFR-positive cancer cells (Table 3). The results of the study demonstrated that this developed system increased the efficiency of drug delivery to the target. Dox loading into BMEs enhanced antitumor activity. Modified BMEs are selective and generally well-tolerated in preclinical models, and serve as an effective tool in EGFR-expressing tumors, demonstrating their feasibility [147]. In a study, aimed to develop a new drug delivery system using milk-derived exosomes. In vitro analyses showed that the developed nanoparticles were more efficient than free DHA. This efficiency could be due to their greater cytotoxic, proapoptotic, and antimetastatic effects (Table 3). The newly developed system was shown to increase drug delivery efficiency. Increased antitumor activity was observed in BMEs loaded with Dox [148]. Another research explained the relationship between exosomes obtained from milk and the miRNAs that play a role in the progression of diffuse large B-cell lymphoma (DLBCL) via cow milk intake. The underlying mechanism of this disease is explained by the excessive stimulation of the PI3K-AKT-mTORC1 pathway, increased BCL6 gene expression, and suppression of BLIMP1/PRDM1. Research shows that regulatory molecules carried by breast milk exosomes, such as miR-148a-3p, miR-155-5p, miR-29b-5p, let-7-5p, and miR-125b-5p, provide epigenetic control over B cell proliferation and differentiation. miRNAs are a novel mechanism in the development of DLBCL. It has been demonstrated that milk exosomes in food may pose a risk [94]. Another study targeted energy metabolism in a lung cancer model. The high expression of ACLY in many cancers targets this enzyme. In this study, folate-functionalized BME was used to increase the efficacy of potassium hydroxide, an ACLY inhibitor. Lung cancer models were induced with urethane. In these models, Exo-KH treatment resulted in a significant reduction in tumor volume and the mRNA levels of lipogenesis-related genes (Table 3). This study highlights the fact that folate-functionalized BME, with CH delivery, is an effective alternative targeted therapy in lung cancer models [149]. Another investigation presented an innovative strategy targeting the tumor microenvironment using a pH- and light-responsive hybrid system designed to deliver therapeutic agents for managing oral squamous cell carcinoma (OSCC). BMEs were used as natural nanocarrier systems. In vitro tests showed that Exo@Dox–EPT1 exhibited a significant cytotoxic effect on HSC-3, CAL-27, and SCC-9 cells; under laser activation, cell viability decreased to 48.0%, 36.6%, and 37.4%, respectively (*p* < 0.05) (Table 3). The findings determined that this formulation creates a powerful synergistic photochemotherapeutic effect by providing pH- and through its ability to release drugs upon light stimulation within the tumor microenvironment, allowing it to function as a multimodal therapeutic system in OSCC treatment [99]. A different approach examined an alternative drug delivery system in NSCLC treatment. The application of PAC in NSCLC has limitations, as observed when the effects of the PAC and BCE formulation were evaluated. These limitations are due to its low solubility and dependence on toxic compounds. In vitro analyses showed increased antiproliferative and colony-inhibiting effects against free PAC with the ExoPAC formulation. Moreover, antitumor effects and bioavailability increased with the ExoPAC formulation. In targeted tissues, FA-functionalized exosomes enhanced accumulation. Tumor growth decreased by 50% when functionalized exosomes were orally administered. Tumor suppression reached 76% when functionalized exosomes were administered intravenously (Table 3). After FA modification, the size of the exosomes slightly increased. In the controlled application of PAC, the FA-ExoPAC system offers a potential alternative delivery system. Elevated antitumor impact and decreased toxicity were observed using this system. The results of this research indicate that BCEs are efficient and viable alternative drug delivery and therapeutic systems for lung cancer models in preclinical conditions [7]. In a study, evaluated the effects of BMEs with KH on ACLY expression. KH is an inhibitor for ACLY. In in vitro studies on NSCLC, Exo-KH formulations were determined to have proapoptotic and antiproliferative effects. The Exo-KH formulation lowers the expression of ACLY, FASN, and IDH1. A suppression of lipid biosynthesis was observed. This study determined the scalable, natural, and biocompatible properties of Exo-KH formulations during in vitro NSCLC model treatment. The results demonstrated the potential effect of Exo-KH formulations, and their safe administration routes [150]. A study examined the effects of fruit-derived compound-loaded BMEs on CRC treatment. Compared to free compounds, the BME formulation showed enhanced selectivity. A significant reduction in tumor number was observed in mouse models when using free and BME formulations (Table 3). The results of the study showed that loaded BMEs suppressed the development of CRC induced by environmental carcinogens under experimental in vitro conditions [151]. In another study, compared the effects of exosomes isolated from camel milk at different times on normal hepatocytes and liver cancer cells. No cytotoxicity was observed on HepaRG in normal hepatocytes. Exosomes isolated from camel milk exhibited cytotoxic effects on HepaRG. Exosomes isolated from camel colostrum exhibited the strongest antitumor effect. The study findings determined that exosomes exhibit selective anticancer effects. They determined that increased apoptosis, the suppression of inflammation, and angiogenesis could be achieved with colostrum-derived exosomes. This study demonstrated that exosomes isolated from colostrum offer a powerful alternative approach to liver cancer treatment. Due to their similar biological components, BMEs may also have the potential to exert similar effects to exosomes isolated from camel milk. However, the effects of exosomes obtained from different milk sources may be context-dependent [9]. Another research investigated the drug delivery effects of exosomes loaded with PAC and functionalized with an iRGD peptide in lung cancer. Analyses revealed that exosomes exhibited antitumor effects in lung adenocarcinoma. The generated exosome system did not exhibit any toxic effects in healthy cells. This study demonstrated that iRGD-functionalized loaded exosomes may have the potential to safely deliver drugs in preclinical lung adenocarcinoma models [131]. Junge et al. investigated the anticancer effects of exosomes isolated from camel milk. It was determined that camel milk exosomes induced dose-dependent cytotoxicity in cancer cell lines. The lack of toxicity in normal Vero cells indicated that this system was selective. The CM-EXO system caused an increase in Bax and caspase-3 expression and induced apoptosis by reducing Bcl-2 levels. All these effects demonstrate that camel milk exosomes are a biocompatible, selective, and safe alternative for cancer treatment. BMEs, whose bioactive components are similar to those of camel milk, may also exhibit these selective cancer effects depending on their biological content [77]. Another investigation aimed to overcome chemotherapy resistance in lung cancer lines. To this end, the therapeutic potential of HA-modified milk exosomes was evaluated. The modified exosomes inhibited tumor growth in in vivo models. Exosomal HA–mEXOs carrying ZNF516 suppressed ABCC5 in PMX-resistant LUAD cells. The inhibition of tumor growth was observed. The developed method can be considered a targeted, compatible, and safe alternative for lung adenocarcinoma [152]. In an experimental design, developed FA-functionalized exosomes. These exosomes aim to overcome resistance to EGFR-tyrosine kinase receptors. The exosomes suppressed c-kit expression. This suppression attenuated the phenotype. The results of the study suggest that this system could eliminate EGFR-TK1 restriction through lung cancer resistance [153]. In another research, studied BRD4 inhibitors. BRD4 inhibitors are emerging as effective anticancer agents that suppress tumor cell proliferation through epigenetic mechanisms. The BRD4 inhibitor was administered orally using camel milk exosomes. In vitro analyses of the study determined that free drug release was 5.4-fold less than that of exosomes. The study results demonstrated that exosomes increase the solubility of the BRD4 inhibitor, providing a potential alternative for epigenetic-based cancer therapies. Due to their structural and compositional similarities, BMEs may similarly improve the pharmacokinetic profile and therapeutic application of epigenetic anticancer drugs under specific pharmacological conditions [154].

## 6. Challenges and Future Perspectives

In complex diseases such as cancer, the potential use of BMEs has been evaluated in recent studies. In cancer, the effects of BMEs on diagnostic, therapeutic, and drug delivery systems were evaluated. High biocompatibility and scalability were observed in exosome effect analyses. Due to technical, biological, and regulatory challenges, the clinical application of exosomes faces difficulties. One of the first difficulties is the storage conditions of the milk from which the exosomes will be isolated. These storage conditions directly affect the quantity, purity, and biological activity of exosomes [158]. When the conditions are not optimal, the membrane integrity of exosomes can be negatively affected, resulting in a decrease in their biological activity. Changes in pasteurization techniques can affect the stability of BMEs. The stability of RNA and protein cargos, which play a role in the antitumor effects of exosomes, may also be affected by these factors [159]. The vast majority of current studies describing the effects of milk-derived exosomes are based on research using raw or minimally processed milk. In contrast, there are no studies that clearly differentiate between UHT-treated milk, raw milk, pasteurized milk, and industrially processed milk. Therefore, the effects of commercial milk processing on exosomes’ integrity and activity remain unclear. Accordingly, caution should be exercised when extrapolating these findings to real-world applications involving commercially processed dairy products [160]. Another challenge in BME applications is loading efficiency and targeting efficiency. Identifying selective targeting strategies is crucial for the stable delivery of BME payloads. Accurately determining the delivery strategy is crucial for the development of therapeutics in cancer cells. Recent studies have demonstrated that BMEs can exert modulatory effects on the tumor microenvironment. Adjusting their controlled release and loading efficiency is crucial for the efficiency of future studies [161]. Exosome engineering allows for antitumor drugs to be delivered to tumor sites, ensuring precise exosome delivery while reducing treatment-related side effects. However, challenges exist in the clinical delivery of these engineered exosomes. One of these challenges is that it is not yet fully understood which sources and modification strategies confer the most effective antitumor activity to exosomes. Furthermore, it has not been clearly determined which engineered exosomes are suitable for different antitumor therapies [162]. The exosomes used in therapeutic applications may face challenges such as insufficient tissue targeting or a short circulatory duration. Engineering applications are targeting these limitations as well [163]. The large-scale production of exosomes is a significant limitation and restricts the clinical applications of engineered exosomes. Strong quality-control standards must be implemented for exosome applications to transition to the clinical level. However, the current methods are insufficient to meet industrial production needs. The cell types that produce high quantities of exosomes most safely have not been clearly identified. Another limitation restricting transfer to clinical applications is the high cost of current exosome isolation methods [164]. Current approaches in cancer immunotherapy face limitations due to the immunosuppressive nature and complexity of the tumor microenvironment. In this context, the use of exosomes holds potential; however, the effectiveness of these approaches is limited by immune evasion mechanisms and the heterogeneous nature of the tumor microenvironment [165]. Another challenge faced by milk-derived extracellular vesicles in the clinical application process is their complex role in cancer, the need for effective antigen delivery, regulatory limitations, and the necessity of standardized production methods. Furthermore, ensuring high targeting specificity and understanding the long-term effects of mEV-based approaches are critical for clinical translation. Therefore, it is necessary to optimize production processes and conduct comprehensive preclinical and clinical studies [102]. Exosomes can be loaded with different therapeutic payloads, such as small molecules, RNA, and CRISPR-Cas systems. Engineered exosomes are reported to offer higher specificity and lower immunogenicity compared to viral vectors and synthetic nanoparticles. In this context, endogenous and exogenous loading strategies and surface functionalization approaches aimed at enhancing targeted delivery are being evaluated alongside advanced loading methods for CRISPR-based applications [166].

Various exosome engineering systems are being developed to overcome these limitations. Lipid-binding and hybrid systems are prominent among these engineering applications. Immunogenicity is another reason for the challenges encountered in exosome applications. BMEs stand out as a potentially better-tolerated alternative following oral administration in preclinical studies. The safety of intravenous delivery methods should be thoroughly investigated due to the potential for hemolytic effects. Intravenously administered exosomes can elicit an immune response [98,159]. The limitations encountered in exosome applications necessitate safety checks before their use in cancer immunotherapy. The molecular mechanisms of BMEs have not been fully elucidated, which is another challenge facing exosome applications. Exosome applications hold preclinical potential due to the effects of miRNA and protein cargo on tumor lines. However, despite their preclinical potential, the effects of exosomes have not been sufficiently validated at the clinical level. The functional characterization of exosome content, GMP-compliant manufacturing technologies, and the development of comprehensive preclinical models to confirm anticancer efficacy should be the primary focus of future studies [167]. In terms of biocompatibility and toxicity, milk-derived exosomes have been identified as biocompatible and are considered safe at the preclinical level. However, studies have not adequately revealed their long-term safety. The possibility of bioaccumulation and the tissue-specific retention of milk-derived exosomes after prolonged repeated administration have not yet been systematically evaluated. There is increasing evidence in studies suggesting that milk-derived exosomes may be largely context-dependent [168]. Therefore, under certain tumor microenvironment conditions, milk-derived exosomes may potentially exhibit pro-tumor effects [169]. Therefore, caution should be exercised in directly transferring safety data from preclinical studies to long-term or clinical applications, and studies should be designed that take these limitations into account. Despite these limitations, BMEs have the potential to provide a safe, scalable, and biocompatible alternative for cancer treatment. Interdisciplinary studies are crucial to translate this potential into clinical success. Production standardization, safety validation, and elucidation of the treatment mechanism are required. BM- and BC-derived exosomes are attracting interest as innovative biocontainers for cancer treatment. Their naturalness, low immunogenic profiles, and payload protection properties make exosomes potential candidates for targeted delivery [98]. It is important to increase the loading capacity of exosomes and conduct future studies focusing on the release mechanisms of hybrid exosome systems [170]. Surface engineering applications could be used to develop strategies through which exosomes selectively target the tumor microenvironment. These strategies could involve ligands or antibodies and could significantly enhance antitumor efficacy [32]. Milk and colostrum exosomes can cross the blood–brain barrier. They are being evaluated as pharmaceutical carriers for glioblastoma and other central nervous system tumors [98]. Moreover, the immunomodulatory and antioxidant effects of BCEs could be combined with immunotherapeutic combinations. This strategy may enable the future programming of tumor immune responses [158].

## 7. Conclusions

BMEs and BCEs are emerging as potentially safe and biologically-based nanotherapeutic systems in cancer treatments. Exosomes possess inherent advantages due to their biological components. These advantages include the ability to pass through the epithelial barrier, aggregate in the tumor microenvironment, and transport therapeutic components. Many preclinical studies have demonstrated reduced side effects using combinations of chemotherapeutic drugs, siRNA, and miRNA. These combinations also demonstrated enhanced antitumor activity. Specifically, the exosome-mediated delivery of agents such as paclitaxel, ARV-825, cannabidiol, and siPDL1 increased drug solubility, cellular uptake, and bioavailability. LF, casein derivatives, and growth factors (IGF, TGF-β, EGF) found in colostrum exosomes facilitate drug delivery. Thanks to their contents, BCEs also exhibit direct anticancer effects through apoptosis, inflammation suppression, and angiogenesis inhibition in in vitro and in vivo studies. Milk exosomes with added folate, hyaluronic acid, and iRGD peptides show enhanced therapeutic efficacy, providing better targeting in lung, liver, and breast cancer models. For this approach to be used in the clinic, manufacturing standards must be finalized, safety data verified, and necessary quality controls completed. Future studies should evaluate the high anticancer potential of BMEs at the preclinical and clinical levels, determine the anticancer effects of BMEs and BCEs at the mechanistic level, and aim to increase the efficiency of exosome applications using safety protocols and standardized methods. BME and BCE, thanks to their bioactive contents, can overcome biological barriers and exhibit anticancer effects. Due to these effects, BME and BCE hold critical potential in cancer treatment and prevention and could serve as alternative treatment methods.

## Figures and Tables

**Table 1:** Conserved miRNAs and lactation stage-dependent differences in milk-derived extracellular vesicles across species.

Component	Species Scope	Colostrum	Mature Milk	Reference
miR-30a-5p	Human + cow + goat (conserved; commonly observed)	Present/commonly observed	Present/commonly observed	[35]
miR-22-3p	Human + cow + goat (conserved; commonly observed)	Present/commonly observed	Present/commonly observed	[35]
miR-26a	Human + cow + goat (conserved; commonly observed)	Present/commonly observed	Present/commonly observed	[35]
Top abundant EV miRNAs overlap (ruminants) (optional strengthening row)	Cow vs. goat EV miRNomes (early lactation)	Reported as shared among top abundant miRNAs (includes miR-26a, miR-30a-5p)	—	[37]
EV miRNA profile differs by lactation stage (stage-difference anchor)	Cow	329 miRNAs differ between colostrum EVs vs. mature milk EVs (RNA-seq)	329 miRNAs differ	[33]
Exosome proteomes differ in lactation stage (quantitative proteomics)	Cow	Colostrum exosomes enriched in proteins linked to immune response and growth	Mature milk differs	[33]

**Table 2:** Summary of proposed mechanisms of action of milk-derived exosomes in cancer models, categorized by mechanism, cargo type, and experimental model, as described in the main text.

Mechanism Category	Specific Mechanism	Exosomal Cargo (as Stated)	Cancer/Model System	Evidence Level
Uptake and carrier potential	Endocytosis, fusion, receptor–ligand interactions	Proteins, lipids, DNA, RNA	General recipient cells	Preclinical
Uptake and carrier potential	FcRn-dependent epithelial transport	miRNA	Caco-2 cell monolayers	Preclinical
Uptake and carrier potential	Glycan-dependent endocytosis	miRNA	Epithelial cells, macrophages	Preclinical
Uptake and carrier potential	Systemic biodistribution after oral intake	miRNA	Intestine, liver, spleen, brain (in vivo)	Preclinical
Uptake and carrier potential	Drug and nucleic acid transport	Small molecules, siRNA, miRNA, paclitaxel	Tumor xenograft models	Preclinical
Apoptosis induction	Caspase-3 activation, mitochondrial dysfunction, ER stress	miR-27b	CRC cells (buffalo milk exosomes)	Preclinical
Apoptosis induction	Gene-level apoptosis regulation	LF-associated cargo	Breast cancer cells	Preclinical
Apoptosis induction	Intrinsic apoptotic pathway activation	Bax increased, caspase-3 increased, Bcl-2 reduced	HepaRG liver cancer cells (camel milk exosomes)	Preclinical
Apoptosis induction	Protein–lipid complex-mediated apoptosis	HAMLET (α-lactalbumin complex)	Glioblastoma, bladder cancer models	Preclinical
Apoptosis induction	Drug-loaded exosome-mediated apoptosis	Curcumin, resveratrol	Cancer cell models (p53-independent)	Preclinical
Apoptosis induction	miRNA-driven apoptosis	miR-34a	Prostate cancer cells (goat milk exosomes)	Preclinical
Modulation of cancer-related inflammation	NF-κB and PI3K/AKT pathway inhibition	miRNAs, TGF-β	LPS-stimulated inflammatory models	Preclinical
Modulation of cancer-related inflammation	Reduction in pro-inflammatory cytokines	IL-6 decreased, TNF-α decreased	LPS-induced systems	Preclinical
Modulation of cancer-related inflammation	Oxidative stress suppression and epithelial protection	Not specified	Intestinal epithelium (animal models)	Preclinical
Modulation of cancer-related inflammation	Intestinal barrier enhancement	TJP1, CLDN1, OCLN decreased	Murine colitis models	Preclinical
Modulation of cancer-related inflammation	Context-dependent pro-tumor inflammatory signaling	TGF-β	Epithelial tumor models	Preclinical
Immunomodulatory effects	Immunosuppressive T-cell modulation	miR-214, IL-10 increased	Tumor immune microenvironment	Preclinical
Immunomodulatory effects	CD8^+^ T-cell apoptosis	Fas/FasL	T cells	Preclinical
Immunomodulatory effects	MDSC differentiation	TGF-β1, PGE2	Tumor microenvironment	Preclinical
Immunomodulatory effects	Antigen presentation enhancement	MHC-I, MHC-II, CD86	Dendritic cell–derived exosomes	Preclinical
Immunomodulatory effects	NK cell and T-cell activation	HSP70-bearing exosomes	Immune cell models	Preclinical
Immunomodulatory effects	Mucosal immune support	Immunoglobulins, immune-related miRNAs	Milk and colostrum exosomes	Preclinical
Immunomodulatory effects	Tumor-supporting immune regulation (risk context)	TGF-β2	Breast cancer cells	Preclinical
Immunomodulatory effects	Species-dependent immune modulation	miRNAs	Camel and buffalo milk exosomes	Preclinical
Exosomal Cargo with Anticancer Potential	Suppression of steroid metabolism-related genes	miR-148a/miR-148a-3p	Cancer cell lines	Preclinical
Exosomal Cargo with Anticancer Potential	Downregulation of AKR1C1, AKR1C2, CYP3A5 expression	Downregulation of AKR1C1, AKR1C2, CYP3A5 expression	miR-148a-3p	Preclinical
Exosomal Cargo with Anticancer Potential	NF-κB signaling suppression	miR-148a	Cancer–inflammation context; intestinal barrier models	Preclinical
Exosomal Cargo with Anticancer Potential	Activation of p53 signaling and apoptosis	miR-34a	Various cancer types	Preclinical
Exosomal Cargo with Anticancer Potential	Regulation of B-cell activation, immune response, and lymphogenesis	miRNAs	Large B-cell lymphoma-related models	Preclinical
Exosomal Cargo with Anticancer Potential	Regulation of metastasis	IGF-derived molecules, TGF-β, casein	Camel milk exosome models	Preclinical
Exosomal Cargo with Anticancer Potential	ROS induction and oxidative stress regulation	LF, κ-casein mRNA	Cancer cell models	Preclinical
Exosomal Cargo with Anticancer Potential	Increased bioavailability and tumor inhibition	Curcumin, DHA, paclitaxel, celastrol	In vitro and in vivo cancer models	Preclinical
Exosomal Cargo with Anticancer Potential	Enhanced efficacy in resistant and in vivo models	ExoPAC, FA-ExoPAC	PAC-resistant cancer models	Preclinical
Exosomal Cargo with Anticancer Potential	Tumor shrinkage via pH- and NIR-responsive release	Exo@Dox–EPT1	OSCC models	Preclinical
Exosomal Cargo with Anticancer Potential	Dose-dependent proliferation inhibition; DNMT1 reduction	miRNA/siRNA	KRAS-mutant lung cancer models	Preclinical

**Table 3:** Preclinical applications of milk- and colostrum-derived exosomes in cancer models.

Study Model	Source of Exosomes	Exosomal Cargo Type/Treatment	Administration Route	Sample Size	Dose/Concentration	Cancer Type/Cell Line	Observed Outcome/Effect	Reference
In vitro (TNBC model)	BME	Doxorubicin-loaded, iRGD-modified, hypoxia-sensitive (iDHRX)	Oral (potential)	NR (number of replicates not specified; 4 TNBC cell lines used)	10 μM iDHRX (50% cell viability in 3D spheroids)	Triple-negative breast cancer (TNBC)	50% reduction in cell viability; specific αvβ3 integrin targeting; no adverse effect on targeting; biocompatible and effective drug delivery	[112]
In vitro (MDA-MB-231 cell line)	BME	Lactoferrin-loaded (exoLF)	In vitro incubation	NR (number of biological replicates not reported)	1 mg/mL exoLF (LF loading: 1 mg/mL LF with 30 µg/mL exosomes; loading efficiency 29.72%)	Breast cancer (MDA-MB-231)	39% late apoptosis; increased Bid gene expression; decreased Bcl-2 expression; high biocompatibility; targeted and effective anticancer delivery	[70]
In vitro	BME	Dihydroartemisinin (DHA)-loaded	In vitro incubation	NR (number of cell lines and biological replicates not reported)	NR (no explicit treatment dose reported)	Cancer cell model (unspecified)	Uniform 100 nm particles; rapid then controlled drug release (pH 7.4/5.5); increased cytotoxicity; increased ROS; decreased mitochondrial membrane potential; decreased migration and colony formation; enhanced bioavailability and anticancer efficacy	[97]
In vitro	BME	Folic acid-functionalized, PAC and 5-fluorouracil (FA-Exo-PAC/5-FU)	Oral exosome delivery concept discussed; in vitro incubation used for assays	NR (replicates not explicitly reported)	PAC: 0.5 µM equivalent, 5-FU: 1 µM equivalent; other IC_50_ values reported for various formulations	Breast cancer	80–100 nm particle size; 82% encapsulation efficiency; 28% loading; sustained 48 h release; decreased IC_50_ values; increased apoptosis and cell internalization; decreased toxicity; enhanced antitumor effect	[144]
In vitro and in vivo	BME	Hyaluronic acid (HA)-coated, miR-204-loaded	In vitro incubation; in vivo intravenous (tail vein) administration	NR (number of animals/cell line replicates not clearly specified)	0.2 nmol miR-204 per dose (6 doses, every 2 days)	Cancer model (unspecified)	High cell and blood compatibility; no systemic toxicity; miR-204 suppressed BCL2 and RAB22A; increased apoptosis; decreased tumor growth; increased doxorubicin sensitivity; decreased chemotherapy resistance	[145]
In vitro	Buffalo milk exosomes	Natural miRNA cargo (miR-148a, miR-15b, miR-27b, miR-125b)	In vitro incubation (cell treatment)	*n* = 3 independent experiments	3 kDa milk extract: 40% (*v*/*v*), 72 h [treatment]	Colorectal cancer (HCT116, HT-29) and pancreatic cancer	miR-27b increased apoptosis, increased mitochondrial ROS, increased lysosomal accumulation; ER stress via PERK/IRE1/XBP1–CHOP; miR-125b increased EMT and metastasis; regulation of proliferation, migration, invasion	[69]
In vitro	BME	Hyaluronic acid-functionalized, doxorubicin-loaded (HA-mExo-Dox)	In vitro incubation	NR (replicate count not reported)	NR (doxorubicin loading dose not explicitly reported)	CD44^+^ cancer cells (MDA-MB-231, A549, MCF-7)	Increased selective Dox uptake; increased apoptosis; decreased cell viability in CD44^+^ cells; no cytotoxicity in CD44^−^ HEK293 cells; enhanced targeting via HA-CD44 interaction	[146]
In vitro	BME	EGFR nanobody (7D12)-functionalized, doxorubicin-loaded	Not specified (in vitro)	*n* = 3 (per experiment)	10 ng/μL (Dox equivalent)	EGFR-positive cancer cells	Increased accumulation in EGFR^+^ cells; decreased toxicity in EGFR^−^ cells; increased drug uptake; increased antitumor efficacy; selective and stable targeted delivery platform	[147]
In vitro	BME	Near-infrared (NIR) dye-loaded (Exo-Glow)	In vitro cell treatment	*n* = 3 (independent experiments)	10 μg ICG-EXO dosage	Breast cancer (MCF-7)	Maintained stability after dye loading; selective tumor labeling; no hemolytic effect; no cytotoxicity; safe and biocompatible platform for bioimaging and theranostic use	[155]
In vitro and in vivo	BME	Dihydroartemisinin (DHA)-loaded (Exo-DHA)	Oral (rats, mice); Intraperitoneal (DTIC control	In vitro: not specified; in vivo: *n* = 5/group	In vitro: 36 µM DHA (Exo-DHA); in vivo: 50 mg/kg DHA (oral)	Melanoma	90–103 nm spherical nanoparticles; increased cytotoxic, proapoptotic, and antimetastatic effects vs. free DHA; decreased Bcl-2, survivin, MMP-9; increased Bax; 2.8× higher bioavailability; increased Cmax (5.431 µg/mL); 3.6× higher AUC_0_–t; reduced hepatotoxicity	[148]
—	Cow milk-derived exosomes	Natural miRNA cargo (miR-148a-3p, miR-155-5p, miR-29b-5p, let-7-5p, miR-125b-5p)	Oral (via milk consumption)	In vitro: 1 × 10^4^ cells/well (MTT); 5 × 10^5^ cells/well (apoptosis) | In vivo: *n* = 5/group	In vitro: 36 µM DHA (Exo-DHA eq.) | In vivo: Exosomes 25 mg/kg; DHA 50 mg/kg; Exo-DHA 50 mg/kg (DHA eq.)	Diffuse large B-cell lymphoma (DLBCL)	Activation of PI3K–AKT–mTORC1 pathway; increased BCL6 expression; decreased BLIMP1/PRDM1; miR-148a-3p increased BCL6 via DNMT1 suppression; miR-125b inhibits PRDM1; potential role of milk exosomes in DLBCL development	[94]
In vivo (urethane-induced lung cancer model)	BME	Folate-functionalized, potassium hydroxide (ACLY inhibitor)-loaded (Exo-KH)	Intraperitoneal (Exo-KH); oral and IP (PK comparison)	6 mice/group (therapy); 12 mice/group (PK)	250 mg/kg (KH or Exo-KH)	Non-small cell lung cancer (NSCLC)	Controlled and sustained release; decreased tumor volume; decreased ACLY, FASN, HMGCR, SREBP1c mRNA levels; confirmed ACLY suppression; targeted and biocompatible metabolic therapy	[149]
In vitro and in vivo	BME	Doxorubicin and anthracycline endoperoxide derivative-loaded (Exo@Dox–EPT1), pH- and light-responsive	Intravenous	In vivo: *n* = 5 mice/group (tumor study); biodistribution: *n* = 4/group	Dox: 0.25 mg/kg (free or NP); exosomes: 60 mg/kg protein	Oral squamous cell carcinoma (HSC-3, CAL-27, SCC-9)	In vitro: decreased cell viability to 48.0%, 36.6%, and 37.4% (*p* < 0.05) under laser; in vivo: decreased tumor volume to 0.05 ± 0.07 cm^3^; strong photochemotherapeutic synergy; sustained tumor fluorescence up to 72 h	[99]
In vitro and in vivo	BC exosomes	Folic acid-functionalized, paclitaxel-loaded (FA-ExoPAC)	Oral and intravenous	Subcutaneous efficacy: *n* = 10/group; orthotopic pilot: *n* = 4/group; orthotopic inhibition: *n* = 10/group; toxicity: *n* = 5/group	Efficacy regimens: PAC 6 mg/kg (p.o. 3×/week; i.v. 1×/week); exosome concentration 50 mg/kg (orthotopic); subcutaneous: oral dosing 3×/week with PAC 6 mg/kg; toxicity: Exo/FA-Exo 60 mg/kg/week oral; PAC 6 mg/kg/week i.p.; ExoPAC 9 mg/kg/week p.o.; FA-ExoPAC 9 or 18 mg/kg/week p.o	Non-small cell lung cancer (A549 and PAC-resistant variants)	Increased bioavailability; increased antiproliferative and colony inhibition vs. free PAC; oral: >50% tumor inhibition; IV: 76% tumor suppression; slight size increase after FA modification; reduced toxicity and enhanced antitumor efficacy	[7]
In vitro	BME	Potassium hydroxide (KH)-loaded, ACLY-targeted (Exo-KH)	Intraperitoneal	In vitro: technical triplicates (*n* = 3); Pharmacokinetics: *n* = 20/group	KH: 250 mg/kg i.p.; PTX: 22.5 mg/kg i.p. (free vs. exosomal forms	Non-small cell lung cancer (A549)	Increased antiproliferative and proapoptotic effects; decreased ACLY, FASN, IDH1 expression; >75% apoptosis; increased ROS and mitochondrial oxidative stress; sustained release (half-life 22.74 h); suppression of lipogenesis and metabolic reprogramming	[150]
In vitro	BME	bcl-2 siRNA-loaded (ExosiBcl-2)	Intravenous	In vitro: ≥3 independent experiments; in vivo: *n* = 5 mice/group	ExosiBcl-2: 1.5 mg/kg siRNA; exosomes alone: 7.5 mg/kg; 5-Fu: 10 mg/kg	Cancer cell lines (unspecified)	Efficient intracellular delivery; decreased proliferation; increased apoptosis; EMT suppression; decreased MAPK, FAK, EGFR, and MMP pathway activity; inhibited migration and invasion; effective siRNA-based gene therapy potential	[156]
In vitro	BM/BC exosomes	Medicinal plant extracts (Apiaceae family: celery, cumin, anise, ajwain) loaded via acid hydrolysis (AH)	In vitro (cell culture exposure via media)	Not explicitly reported (HPLC measurements repeated in triplicate; cells seeded per well stated)	Cell assay: extracts 0–1 mg/mL (later max 500 µg/mL) for 68–70 h; exosome loading: 1 mL extract (10 mg/mL) + 20 mg exosomal protein (PBS), incubate 30 min, then wash ×3	Breast cancer	Increased antiproliferative and cytotoxic effects vs. free extracts; increased bioactivity after AH; increased loading efficiency up to 8× (hydrophobicity-dependent); enhanced bioavailability and anticancer efficacy	[157]
In vitro and in vivo (Apc^Min/+ mouse model)	Milk-derived exosomes (UN source)	Fruit-derived anthocyanidins-loaded (ExoAnthos)	In vitro: cell culture exposure; In vivo: oral gavage	In vitro: 3.0 × 10^3^ cells/well; in vivo: *n* = 5 mice total (male *n* = 2, female *n* = 3)	In vitro: Anthos/ExoAnthos 25–200 µmol/L (24–72 h); in vivo: ~8.6 mg/kg/day, oral, 3 days/week for 4 weeks	CRC	Decreased cell viability (dose-dependent); increased selectivity index; decreased number of colon tumors; restored phase I/II enzyme balance; suppression of CRC development linked to bacterial toxins and carcinogens	[151]
In vitro	Camel milk-derived exosomes (colostrum, early, mid-lactation)	Natural cargo (LF, κ-casein, bioactive molecules)	In vitro exposure	Cells: 1 × 10^4^ cells/well (96-well format)	HepaRG: 0–100 µg/mL; THLE-2: 0–500 µg/mL (24 h; IC_50_-based dosing for mechanistic assays)	Liver cancer (HepaRG) vs. normal hepatocytes (THLE-2)	Colostrum exosomes: strongest antitumor effect; increased Bax, increased caspase-3, decreased Bcl-2; decreased TNFα, NFκB, TGFβ1, COX-2, VEGF; highest DNA damage; IC_50_ = 20.6 µg/mL; selective cytotoxicity toward cancer cells; particle size 30–100 nm; highest yield ≈ 410 mg/L	[9]
In vitro	Milk-derived exosomes	Paclitaxel-loaded, iRGD-functionalized	In vitro (cell culture exposure)	Not explicitly reported	Not explicitly reported	Lung adenocarcinoma	Significant antitumor effect; no cytotoxicity in normal cells; increased intracellular PAC accumulation; effective penetration into 3D tumor spheroids; high selectivity and biocompatibility	[131]
In vitro	Camel milk-derived exosomes (CM-EXOs)	Natural cargo	In vitro (cell culture exposure via media)	Not explicitly reported (cells seeded at 1 × 10^4^ cells/well; multiple dose groups defined by IC_50_ fractions)	CM-EXOs: 0–200 µg/mL (HepG2, CaCo2) and 0–500 µg/mL (Vero); mechanistic assays at ¼ IC_50_, ½ IC_50_, IC_50_ for 24 h	Liver cancer and colorectal cancer (Caco-2)	Dose-dependent cytotoxicity; strongest effect in Caco-2 cells; no toxicity in Vero cells; increased Bax and caspase-3, decreased Bcl-2; increased ROS and oxidative stress; decreased Nrf2/HO-1; apoptosis and oxidative stress-mediated cell death; selective and biocompatible system	[77]
In vitro and in vivo	Milk-derived exosomes	Hyaluronic acid (HA)-modified, carrying ZNF516	In vitro: cell culture exposure. In vivo: intravenous injection (xenograft model)	In vitro: cell culture exposure. In vivo: intravenous injection (xenograft model)	In vitro: HA–mEXOs 3.75–120 μg/mL; EXO uptake studies 30 μg/mL; PMX 0.001–100 μM. In vivo: PMX 100 mg/kg; HA–mEXOs or mEXOs 25 mg/kg, 3×/week	Lung adenocarcinoma (PMX-resistant LUAD cells)	Increased cellular uptake; restored PMX sensitivity; decreased proliferation, invasion, and metastasis; decreased tumor growth in vivo; HA–mEXOs suppressed ABCC5; targeted, biocompatible, and safe nanotherapeutic platform	[152]
In vitro and in vivo	Milk-derived exosomes	Folic acid-functionalized, siRNA-loaded targeting c-kit (FA-mExo-siRNA-c-kit)	In vivo: IV (tail vein) for biodistribution, toxicity, and metastasis; intratumoral for mExo treatment; oral gavage for gefitinib. In vitro: cell culture exposure.	Biodistribution: BALB/c nude *n* = 4/group. Xenograft: BALB/c nude *n* = 5/group (5 groups). Metastatic liver model: *n* = 5/group (5 groups). (In vitro: experiments stated triplicate for qRT-PCR)	Biodistribution: DiR-exosomes 60 mg/kg (IV, single). Toxicity: FA-mExo-siRNA-c-kit 60 mg/kg (IV, daily ×7). Xenograft therapy: Gefitinib 5 mg/kg (gavage) ± FA-mExo 60 µg/kg (intratumoral). Metastasis: FA-mExo (±siRNA) 60 µg/kg (IV, qod ×12) + gefitinib 5 mg/kg (gavage ×12).	Gefitinib-resistant lung cancer	Decreased c-kit expression; suppression of EMT and stem-like phenotype; decreased tumor growth; increased survival with gefitinib co-administration; effective strategy against EGFR-TKI resistance	[153]
In vitro and in vivo	Milk-derived exosomes	siPDL1-loaded, M2pep- and 7D12-functionalized (7D12-mExo-M2pep-siPDL1)	Intravenous tail injection	In vivo therapy: orthotopic MC38 model 6 groups, *n* = 6 mice/group. Biodistribution: number of mice not specified. (In vitro: no explicit n; multiple assays performed; loading efficiency via fluorescence standard curve)	In vivo: IV tail vein injection (biodistribution + therapy). In vitro: cell incubation/coculture assays (macrophages, cancer cells)	EGFR^+^ tumor models	Successful PD-L1 silencing; M2→M1 macrophage conversion; 90% tumor growth inhibition; decreased liver metastasis; increased survival; immunoactive tumor microenvironment; effective TAM-targeted immunotherapy platform	[133]
In vitro and in vivo	Camel milk-derived exosomes (CME)	BRD4 inhibitor ARV-825-loaded (ARV-825–CME)	Oral	Tumor uptake (mouse): 3 groups, *n* = 3/group PK (rat): *n* = 5/group Intestinal distribution: *n* = 3/timepoint In vitro: ≥3 repeats (often triplicate)	5 mg/kg (rats, PK); in vitro 150 µg/mL	Cancer cell lines (SF8628, H1975R)	42.75% encapsulation; 136.8 nm size; –32.75 mV zeta potential; 5.4× faster release vs. free drug; 1.5–2× lower IC_50_; 2.55× higher Cmax; 5.56× higher AUC; increased solubility, absorption, and therapeutic efficacy	[154]

## Data Availability

No new data were created or analyzed in this study. Data sharing is not applicable to this article.
